# Isomerization of Cyclooctadiene to Cyclooctyne with a Zinc/Zirconium Heterobimetallic Complex

**DOI:** 10.1002/anie.201601758

**Published:** 2016-04-13

**Authors:** Michael J. Butler, Andrew J. P. White, Mark R. Crimmin

**Affiliations:** ^1^ Department of ChemistryImperial College LondonSouth KensingtonLondonSW7 2AZUK

**Keywords:** cyclooctynes, diene isomerization, heterobimetallic complexes, hydrides, zirconium

## Abstract

Reaction of a zinc/zirconium heterobimetallic complex with 1,5‐cyclooctadiene (1,5‐COD) results in slow isomerization to 1,3‐cyclooctadiene (1,3‐COD), along with the formation of a new complex that includes a cyclooctyne ligand bridging two metal centers. While analogous magnesium/zirconium and aluminum/zirconium heterobimetallic complexes are competent for the catalytic isomerization of 1,5‐COD to 1,3‐COD, only in the case of the zinc species is the cyclooctyne adduct observed.

Despite decades of research into alkene isomerisation,^1^ the conversion of dienes into alkynes is, to the best of our knowledge, unknown. A related reaction, alkene‐to‐alkyne dehydrogenation, has only limited precedent.^2^ The paucity of data can be explained by considering the thermodynamics of isomerization. From gas‐phase calorimetry measurements, the Δ_f_ *H* ^o^ values for 1,3‐butadiene and 2‐butyne are 26.7±0.2 and 34.7±0.2 kcal mol^−1^ respectively.^3^ Diene‐to‐alkyne isomerization becomes increasingly unfavorable within small or medium carbocycles; the product would be expected to incorporate significant ring strain (Figure 1).^4^ Consistent with these data, the microscopic reverse reaction, alkyne‐to‐diene isomerisation, can be catalyzed by rhodium/BINAP,^5^, ^6^ ruthenium hydride,^7^ or gold(I) complexes.^8^

**Figure 1:**
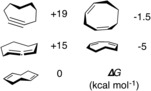
Relative Gibbs free energies (298.15 K) of isomers of C_8_H_12_ (B3LYP functional and 6,311G+(d,p) basis set).

Herein, we show that the isomerization of cyclooctadiene to cyclooctyne is possible within the coordination sphere of an unusual Zn/Zr heterobimetallic complex. The favorable binding of cyclooctyne provides a thermodynamic driving force for the isomerization. The balance of the stabilizing interactions that allow trapping of the cyclooctyne on the bimetallic complex is underscored by control reactions in which Mg/Zr, Al/Zr, and Zr/Zr bimetallic complexes all fail to result in alkyne trapping, but rather give the expected isomerization of 1,5‐cyclooctadiene (1,5‐COD) to 1,3‐cyclooctadiene (1,3‐COD).

The heterobimetallic complexes **M⋅Zr** (M=Zn, Mg, Al(H)^9^) are products of the simple addition of the known hydrides (Figure 2). Following isolation, their solution and solid‐state structures were confirmed and correlated by single‐crystal X‐ray diffraction, multinuclear and variable‐temperature (VT) NMR, DFT studies, and CHN analysis. Although there is limited precedent for complexes containing Zr−H−Mg groups,^10^ those containing a Zr−H−Zn moiety are unknown. The Zr−Zn distance of 2.8866(9) Å is in excess of the sole example of a Zr−Zn bond in [Cp_2_Zr(ZnR)_2_] (R=C_6_H_3_‐2,6‐(2,4,6‐iPr_3_C_6_H_2_)_2_), which was found to be 2.7721(7) Å.^11^ The longer intermetallic distance for **Mg⋅Zr** (3.090(3) Å) reflects the increase in the covalent radius of Mg with respect to that of Zn (1.41 and 1.22 Å, respectively).^12^ The structures are consistent with the molecular orbital arguments originally proposed by Ballhausen and Dahl for complexes of the form [Cp_2_MH_3_].^13^

**Figure 2:**
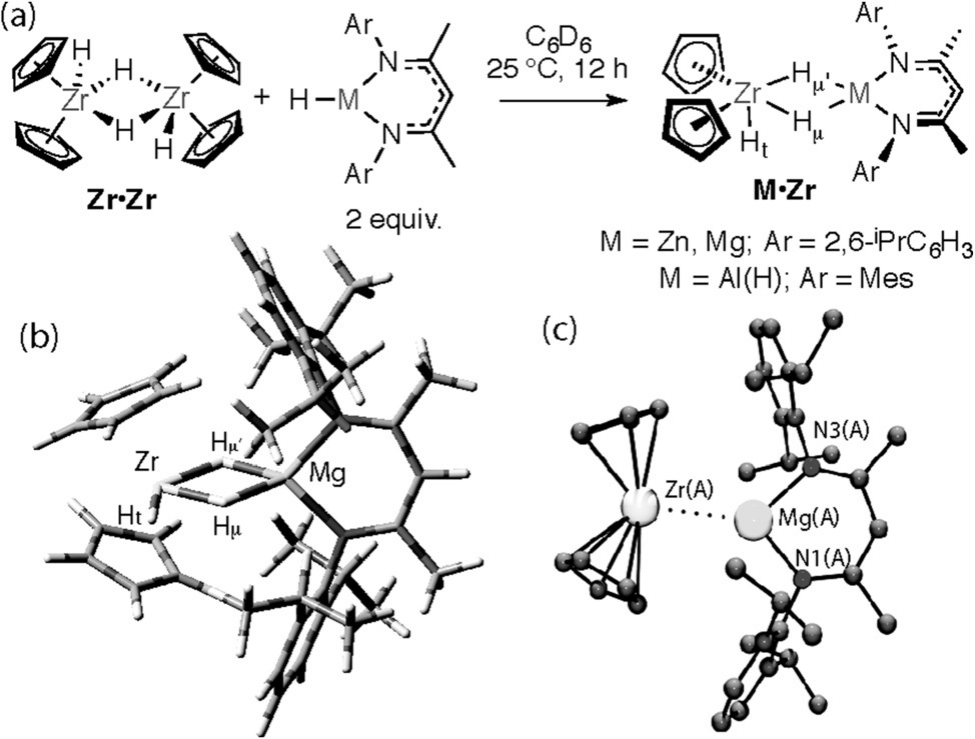
a) Synthesis of **M⋅Zr**. b) The calculated structure of **Mg⋅Zr**. c) The crystal structure of **Mg⋅Zr**. Selected bond lengths [Å] and angles [°]: Zr(A)−Mg(A) 3.090(3), Mg(A)−N1(A) 2.057(8), Mg(A)−N3(A) 2.036(8), N3(A)‐Mg‐N1(A) 91.9(4).

The bridging hydrides H_*μ*_ and H_*μ*′_ in **Mg⋅Zr** are chemically and magnetically inequivalent, and at 298 K in [D_8_]toluene they resonate at *δ*=−2.00 (dd, ^2^ *J*_H‐H_=13.0, 6.6 Hz) and −2.87 (dd, ^2^ *J*_H‐H_=6.6, 6.6 Hz) ppm, respectively.^14^, ^15^ Both couple to the terminal hydride (H_*t*_) found at *δ*=1.55 (dd, ^2^ *J*_H‐H_=13.0, 6.6 Hz) ppm. While VT NMR experiments on **Mg⋅Zr** or **Zn⋅Zr** in [D_8_]toluene (273–353 K) provide no evidence for disintegration of the heterobimetallic complexes in solution, site exchange between the terminal hydride (H_*t*_) and the distal bridging hydride (H_*μ*′_) was observed at all temperatures. In contrast, **Al⋅Zr** forms reversibly and readily establishes an equilibrium with the parent hydrides.^9^

The reaction of **Zn⋅Zr** with 1,5‐COD in C_6_D_6_ at 80 °C resulted in more than 50 % conversion of the hydrocarbon within 24 h and formation of 1,3‐COD (22 % based on 1,5‐COD), cyclooctene (80 % based on **Zn⋅Zr**), the heterobimetallic complex **1** (85 % based on **Zn⋅Zr**), and trace cyclooctane (Scheme 1).

**Scheme 1:**
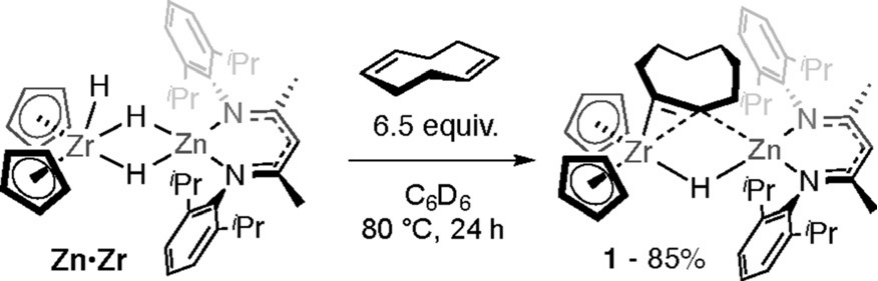
Isomerization of 1,5‐cyclooctadiene with **Zn⋅Zr**.

Over the course of the reaction, the hydride resonances of **Zn⋅Zr** observed at *δ*=−1.09 (d, ^2^ *J*_H‐H_=8.3 Hz), −2.07 (d, ^2^ *J*_H‐H_=6.3 Hz) and 1.80 (dd, ^2^ *J*_H‐H_=8.3, 6.3 Hz) ppm slowly decreased in intensity and were replaced by a new resonance at *δ*=−1.22 (s) ppm. The protons adjacent to the unsaturated carbon–carbon bond in **1** are observed as a series of overlapping multiplets at *δ*=3.13–3.18 ppm, while the ^13^C NMR resonances of the alkyne appear at *δ*=111.9 and 184.3 ppm. Following a preparative‐scale experiment, **1** was isolated as single crystals from an *n*‐hexane solution at −35 °C. While the on‐metal conversion of cyclooctyne into either 1,2‐cyclooctadiene or 1,3‐cyclooctadiene is known, to the best of our knowledge, the conversion of a diene into an alkyne has not been reported.^16^ Although the related bimetallic complexes **Mg⋅Zr**, **Al⋅Zr**, and [Cp_2_Zr(*μ*‐H)(H)]_2_ consistently gave mixtures of 1,3‐COD, cyclooctene, and cyclooctane from 1,5‐COD, only the zinc analogue demonstrated the formation of a metal‐bound cyclooctyne.

A single‐crystal X‐ray diffraction experiment with **1** revealed an unusual bonding mode for the cyclooctyne that may provide insight into the dependence of the reactivity on the main‐group fragment (Figure 3).^17^ The zirconium and zinc metal centers are bridged by not only the alkyne ligand but also a single hydride. The Zr−C bond lengths are consistent with the expected asymmetry of the alkyne bridge and take values of 2.176(1) Å and 2.444(2) Å for terminal and bridging carbons, respectively. The Zr−Zn distance of 2.7943(3) Å is approximately 0.1 Å shorter than that observed in the parent complex **Zn⋅Zr**. The Zn−C bond length of 2.198(2) Å is slightly longer than the 1.9–2.1 Å range established for terminal Zn−C σ‐bonds,^18^ but shorter than the range of 2.2–2.3 Å reported for strongly π‐coordinated alkene complexes of Zn^II^.^19^ The short C−C bond length of 1.308(3) Å and obtuse C‐C‐C bond angles of 129.0(2) and 133.9(2)° support the formulation as a coordinated cyclooctyne.^20^, ^21^, ^22^, ^23^

**Figure 3:**
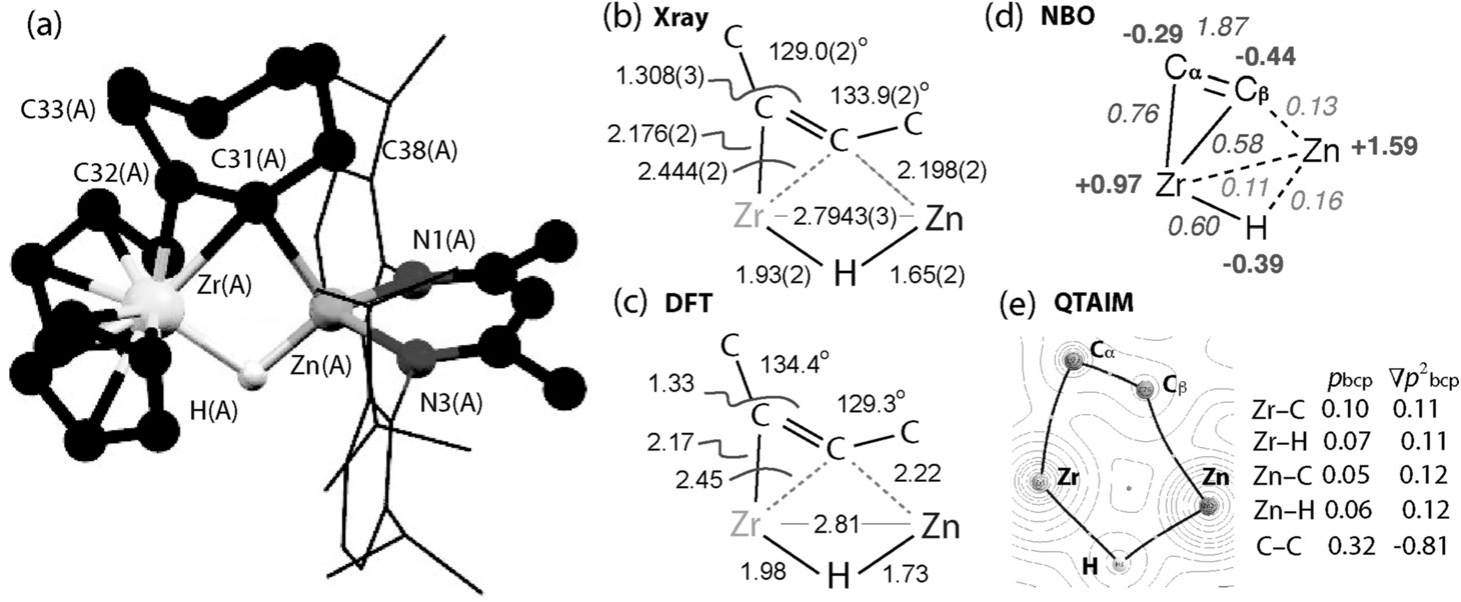
a) The crystal structure of molecule A within the unit cell of **1**. b) Selected bond lengths [Å] and angles [°] from the molecule A within the unit cell. c) Selected bond lengths [Å] and angles [°] from the calculations (ωB97x functional and hybrid 6,311G+(d,p)/SDD basis set). d) NPA charges (bold) and Wiberg Bond indices (italics) represented on **1**. e) Electron‐density contour plot with an overlaid calculated structure from QTAIM presented in the {ZrHZn} plane; the dots show bond‐critical or ring‐critical points.

There is precedent for the structure of **1**: the synthesis of related complexes was pioneered by Buchwald, Erker, and others as part of studies into planar tetracoordinate carbon species of the form R_2_CM^1^M^2^ (M^1^=Zr, M^2^=Zr, Al, Ga, B).10b, 24, 25 Initial attempts to displace the cyclooctyne fragment from **1** with excess trimethylphosphine at 80 °C led to no change in the reaction mixture. Despite this finding, complex **1** catalyzes the isomerization of 1,5‐COD to 1,3‐COD at 5 mol % loading and 80 °C, albeit extremely slowly over a period of 4 weeks.

Calculations employing the ωB97X functional and a hybrid 6,311G+(d,p)/SDD basis set accurately reproduce metrics obtained from X‐ray crystallography. Both NBO (including second‐order perturbation theory^26^) and QTAIM analysis are consistent with the formulation of **1** as a metallacyclopropene complex (Figure 3). While the two computational approaches assign different importance to the Zn−C_β_ and Zr−C_β_ interactions, neither support a Zr^II^ valence‐bond description. In combination, the data suggest that the hydrocarbon ligand of **1** is best described as a slipped metallacyclopropene.25e Fragment analysis of **1** allows quantification of the important factors that stabilize this complex. Consideration of the distortion energies of the alkyne (*E* ^1^_dist_=39 kcal mol^−1^) and bimetallic complex (*E* ^2^_dist_=16 kcal mol^−1^) fragments along with the energy of alkyne binding (*E*_bind_=−119 kcal mol^−1^) reveals that the deformation energies are aptly compensated for by the large binding energy (Figure S24 in the Supporting Information). Only in the presence of this binding event does the isomerization reaction become thermodynamically favorable. Further calculations show that transfer hydrogenation of 1,5‐COD to form cyclooctene is not a requisite for the transformation, hence ligand exchange from **Zn⋅Zr**+cyclooctyne→**1**+H_2_ is calculated to be exergonic with Δ*G*°_rxn_=−22 kcal mol^−1^.

While the mechanism of diene‐to‐alkyne isomerization remains somewhat opaque, two important control experiments are consistent with a chain‐walking process and not hydrogenation followed by dehydrogenation. Hence, while reaction of **Zn⋅Zr** with excess 1,3‐COD gives **1** in 90 % yield by NMR spectroscopy, under identical conditions, reaction with cyclooctene leads to decomposition of the heterobimetallic complex with no evidence for the formation of **1**.

We are continuing to study the reactivity of these heterobimetallic complexes and the mechanism of diene‐to‐alkyne isomerisation.

## Supporting information

As a service to our authors and readers, this journal provides supporting information supplied by the authors. Such materials are peer reviewed and may be re‐organized for online delivery, but are not copy‐edited or typeset. Technical support issues arising from supporting information (other than missing files) should be addressed to the authors.

Supplementary
