# Supplementary material for: Isomerization of Cyclooctadiene to Cyclooctyne with a Zinc/Zirconium Heterobimetallic Complex
Source: Angew Chem Int Ed Engl. 2016 Apr 13;55(24):6951–3. doi: 10.1002/anie.201601758 (PMC5074263; doi:10.1002/anie.201601758)
Supplement: Supplementary file 1 — Supplementary [file ANIE-55-6951-s001.pdf]

Angewandte Chemie
Eine Zeitschrift der Gesellschaft Deutscher Chemiker

Supporting Information

Isomerization of Cyclooctadiene to Cyclooctyne with a Zinc/Zirconium Heterobimetallic Complex

*Michael J. Butler, Andrew J. P. White, and Mark R. Crimmin**

anie_201601758_sm_miscellaneous_information.pdf

Table of Contents

1	GENERAL EXPERIMENTAL	S3
2	SYNTHESES AND CHARACTERISATION OF THE HETEROBIMETALLIC HYDRIDES	S4
3	CATALYTIC ISOMERISATION OF CYCLOOCTA-1,3-DIENE, AND THE SYNTHESIS OF 1	S16
4	DFT STUDIES	S22
5	X-RAY DATA	S28
6	MULTINUCLEAR NMR DATA	S34
7	Z MATRICES	S38

1 GENERAL EXPERIMENTAL

All manipulations were carried out using standard Schlenk-line and glovebox techniques under an inert atmosphere of argon or dinitogen. A MBraun Labmaster glovebox was employed, operating at < 0.1 ppm O_2 and < 0.1 ppm H_2O. Solvents were dried over activated alumina from an SPS (solvent purification system) based upon the Grubbs design and degassed before use. Glassware was dried for 12 h at 120 °C prior to use. Benzene-d_6 and toluene-d_8 were stored over molecular sieves and distilled prior to use. NMR-scale reactions were conducted in J. Youngs tap tubes and prepared in a glovebox. All heating mentioned was done using silicone oil baths. ^{1}H and ^{13}C, NMR spectra were obtained on BRUKER 400 MHz or 500 MHz machines unless otherwise stated; all peak intensities are derived internal standard peak with values quoted in ppm. Data was processed using the MestReNova software.

1.1 MATERIALS

$LiAlH_4$ was purified by extraction into THF (10 g in approximately 50 ml), followed by filtration and removal of the solvent under reduced pressure. The mixture was heated to 45 °C to remove the last traces of solvent and the colourless solid isolated proved soluble in THF and Et_2O. Zirconocene dihydride ($[Cp_2ZrH(\mu\text{-}H)]_2$ or here, **Zr•Zr**) was prepared by the procedure of Wailes and Weigold.[1] The syntheses of the proligand *BDI*H and the series of *β*-diketiminate-stabilised magnesium, zinc and calcium hydrides was achieved using literature procedures.[2] Ferrocene was sublimed before use. Hydrocarbons were dried over activated molecular sieves and freeze-pump-thaw degassed before use. All other other chemicals were purchased from Sigma Aldrich and used without purification unless stated.

---

[1] P. C. Wailes, H. Weigold, A. P. Bell, *J. Organomet. Chem.* **1972**, *43*, C29.
[2] (a) A. E. Nako, S. J. Gates, A. J. P. White, M. R. Crimmin, *Dalton Trans.* **2013**, *42*, 15199. (b) J. Spielmann, D. Piesik, B. Wittkamp, G. Jansen, S. Harder, *Chem. Commun.* **2009**, 3455. (c) S. J. Bonyhady, C. Jones, S. Nembenna, A. Stasch, A. J. Edwards, G. J. McIntyre, *Chem. Eur. J.* **2010**, *16*, 938.

2 SYNTHESES AND CHARACTERISATION OF THE HETEROBIMETALLIC HYDRIDES

2.1 SYNTHESES

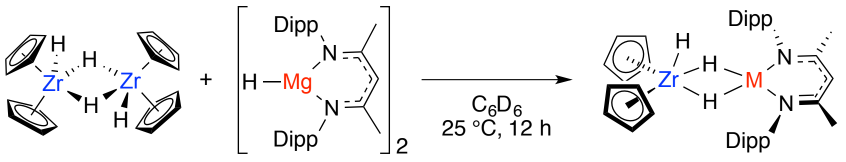

Scheme S1. Synthesis of $[(DippNCMe)_2CH_2]Mg(\mu\text{-}H)_2HZrCp_2$, **Mg•Zr** (Dipp = 2,6-di-iso-propylphenyl)

Synthesis of Mg•Zr: In a glovebox, the magnesium(II) hydride (298 mg, 0.671 mmol, 1 equiv.) was added to a 20 mL scintillation vial and dissolved in 15 mL dry toluene. The zirconocene dihydride (150 mg, 0.336 mmol, 1 equiv.) was weighed and added to the flask as a solid. The reaction mixture was stirred for 48 h. The solvent was removed under reduced pressure to give a glassy oil of yellowish hue. Dissolving the crude material in ca. 20 mL of *n*-hexane gave a yellow solution, which was filtered and concentrated to ca. 5 mL. Storage of this solution a -35 °C resulted in crystallization of the product ***Mg•Zr*** (254 mg, 0.369 mmol, 55%). ^{1}H NMR (400 MHz, 298 K, benzene-d_6) δ 7.12-7.00 (m, 6H, H_{Aryl}), 4.99 (s, 10H, Cp_2), 4.94 (s, 1H, C_β), 3.27 (hept, $^{3}J_{H\text{-}H}$ = 6.6 Hz, 4H, C*H*Me_2), 1.46 (d, $^{3}J_{H\text{-}H}$ = 6.6 Hz, 6H, C*Me*HMe'), 1.13 (d, $^{2}J_{H\text{-}H}$ = 6.6 Hz, 6H, CMeH*Me'*), -1.87 (dd, $^{2}J_{H\text{-}H}$ = 13.0, 6.6 Hz, 1H), -2.80 (dd, $^{2}J_{H\text{-}H}$ = 6.6, 6.6 Hz, 1H). ^{13}C NMR (101 MHz, 298 K, benzene-d_6) δ 169.33 (N–C_α), 144.66 (C_{ipso}), 142.49 (C_o), 126.36 (C_p), 124.46 (C_m), 97.41 (Cp_2), 94.90 (C_β), 28.54 (C_{iPr}), 24.43 (CH*Me*$_2$), 24.34 (CH*Me*), 23.85 (*Me*); FTIR *v* (cm^{-1}): 2959 s, 2868 s, 1551 s, 1523 s, 1436 s, 1403 s, 1317 s, 1261s, 1179 s, 1100 s, 1017 s, 936 s, 855 s, 795 s. Elemental analysis calc. for $C_{39}H_{54}MgN_2Zr$: C, 70.29%; H, 8.17%; N, 4.20%; found C, 70.11%, H, 8.00%; N, 4.36%

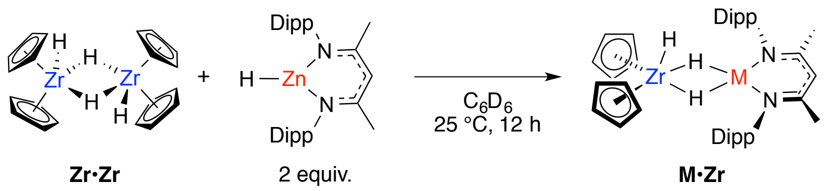

Scheme S2. Synthesis of $[(DippNCMe)_2CH_2]Zn(\mu\text{-}H)_2HZrCp_2$, **Zn•Zr** (Dipp = 2,6-di-iso-propylphenyl)

Synthesis of Zn•Zr: In a glovebox, the zinc(II) hydride (325 mg, 0.671 mmol, 2 equiv.) was added to a dry schlenk flask and dissolved in dry toluene to make a 0.056 M solution. The zirconocene dihydride (150 mg, 0.336 mmol, 2 equiv.) weighed and added to the flask as a solid. The reaction mixture was stirred overnight, and the resulting solution was filtered and concentrated to approximately 3 mL. The product was crystallized from this solution over two days at -20 °C. Large, colorless, cubic crystals of ***Zn•Zr*** were obtained (179 mg, 0.253, 38%). ^{1}H NMR (400 MHz, 298 K, benzene-d_6) δ 7.12 – 7.09 (m, 6H, H_{Aryl}), 4.95 (s, 1H, H_β), 4.94 (s, 10H, Cp_2), 3.36 (br, 4H, C*H*Me_2), 1.95 – 1.91 (dd, $^2J_{H\text{-}H}$ 8.3, 6.3 Hz, 1H, H_t), 1.47 (br, 6H, C*Me*HMe'), 1.14 (br, 6H, CMeH*Me'*), -1.08 (d, $^2J_{H\text{-}H}$ = 8.3 Hz, 1H, H_μ), -2.06 (d, $^2J_{H\text{-}H}$ = 6.3 Hz, 1H, $H_{\mu'}$). ^{13}C NMR (101 MHz, 298 K, benzene-d_6) δ 167.46 (C_α), 146.09 (C_{ipso}), 142.51 (C_o), 126.41 (C_p), 124.52 (C_m), 97.97 (Cp_2), 94.64 (C_β), 28.39 (C_{iPr}), 24.47 (CH*Me*$_2$), 24.29 ($CHMe_2$), 24.05 (*Me*); FTIR v (cm^{-1}): 2959 s, 2866 s, 1622 s, 1549 s, 1519 s, 1460 s, 1436 s, 1402 s, 1363 s, 1314 s, 1259 s, 1174 s, 1103 s, 1063 s, 1015 s, 934 s, 792 s. Elemental analysis calc. for $C_{39}H_{54}ZnN_2Zr$: C, 66.21%; H, 7.69%; N, 3.96%; found C, 66.09%, H, 7.49%; N, 3.98%

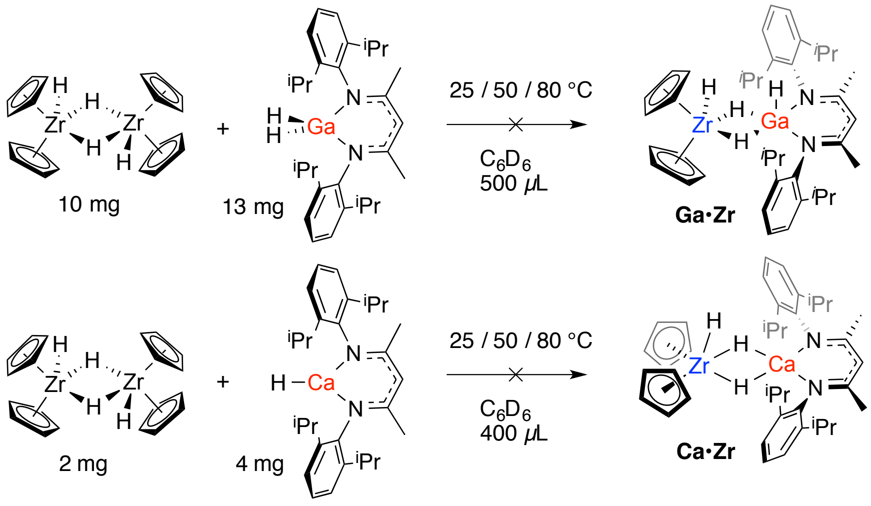

Scheme S3. Attempted synthesis of **Ca•Zr** and **Ga•Zr** (Dipp = 2,6-di-iso-propylphenyl)

No formation of **Ga•Zr** was witnessed in a test reaction using *β*-diketiminate-supported gallium hydride. Treatment of *β*-diketiminate-supported calcium hydride with zirconocene dihydride gave ligand redistribution about the Ca centre at room temperature to form $[(DippNCMe)_2CH_2]_2Ca$ and the putative **Ca•Zr** heterobimetallic could not be isolated.

2.2 *Variable Temperature ^{1}H NMR STUDIES*

Figure S1. Important ^{1}H NMR chemical shift data for complexes **Zn•Zr** and **Mg•Zr** in toluene-d_8 at room temperature. The terminal hydride was confirmed and assigned in 1D NOESY experiments. Analogous data for **Al•Zr**, including VT and 1D NOESY experiments can be found in a previous publication by the group.[3]

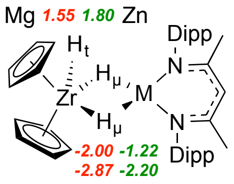

	Splitt.		$^2J_{H-H}$ /Hz	
H_t	dd	dd	13.0, 6.6	8.3, 6.3
H_μ	dd	d	13.0, 6.6	8.3
H_μ'	dd	d	6.6	6.3

Figure S2. Hydride T_1 Relaxation Times

Hydride	T_1	
	**Zn•Zr**	**Mg•Zr**
H_t	950 ms	700 ms
H_μ	570 ms	530 ms
$H_{\mu'}$	1.1 s	1.2 s

400 MHz, toluene-d_8 , 298 K

[3] S. Yow, S. J. Gates, A. J. P. White, M. R. Crimmin, *Angew. Chem., Int. Ed.* **2012**, *51*, 12559.

Figure S3. VT NMR of **Mg•Zr** between 193–373 K: cooling reveals broadening of the hydride signals H_{μ} (δ = -2.02 at 273 K) and $H_{\mu'}$ (δ = -2.93 at 273 K) under 223 K. Broadening and splitting out of the Nacnac ligand aryl substituents occurs below 243 K.[4]

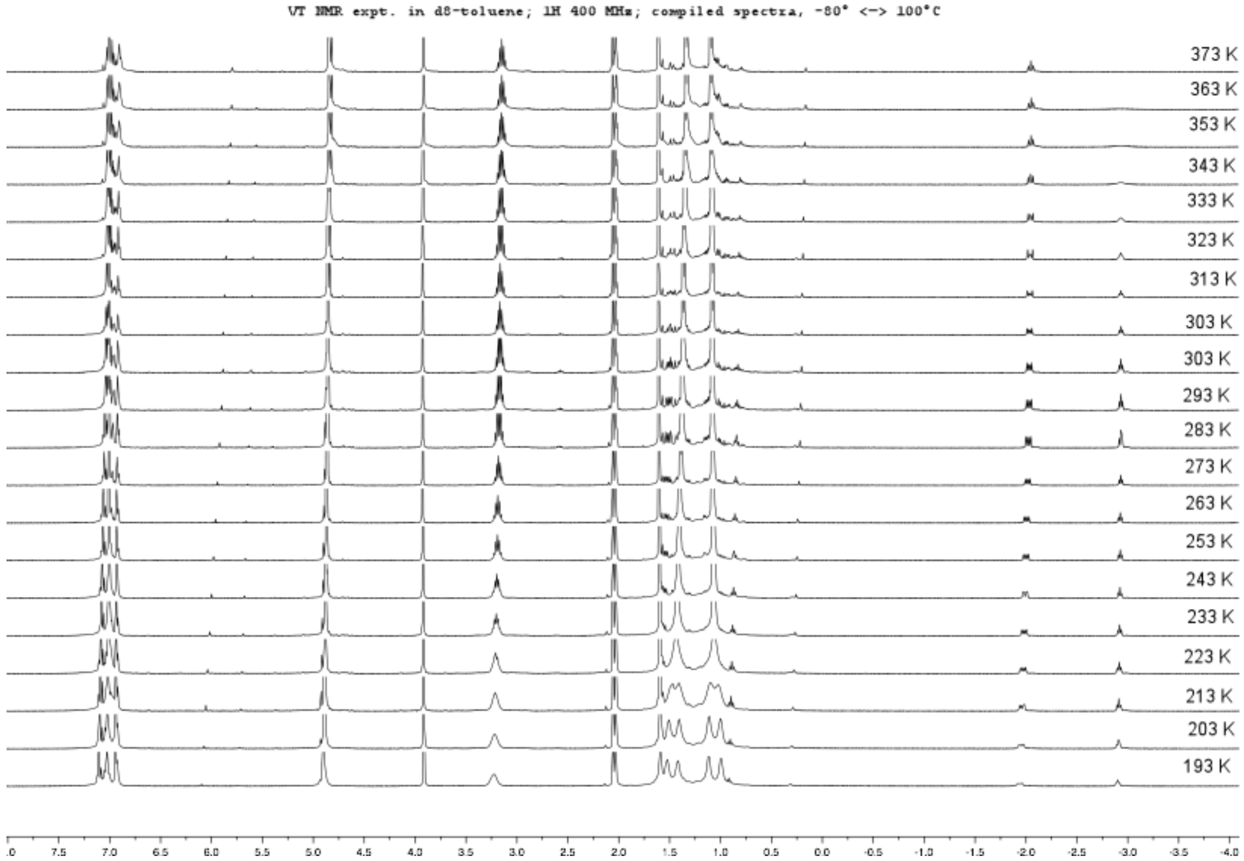

[4] The coalescence temperature of the observed exchange processes of the metal hydrides appears dependent on the trace impurity profile, and different batches of **Mg•Zr** showed the same chemical exchange process but operating at lower temperature. We report here the upper limit at which the chemical exchange between H_t and $H_{\mu'}$ is observed.

Figure S4. VT NMR of **Zn•Zr** between 193–373 K: the chemical exchange process between the H_t and $H_{\mu'}$ leads to broadening above room temperature. T_C = 373 K, with a concomitant collapse of the H_μ doublet to form a triplet. Broadening and splitting out of the Nacnac ligand aryl substituents occurs below 323 K.

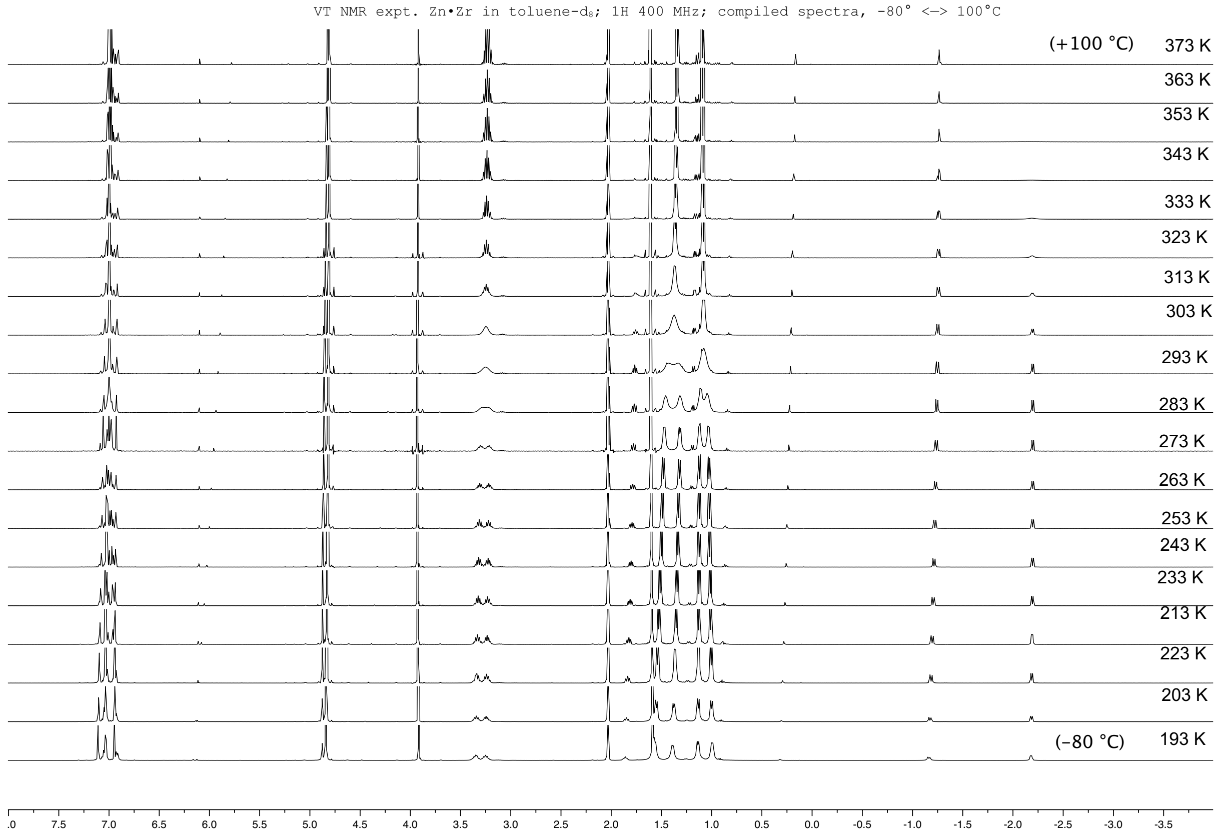

VT NMR data was treated by line shape analysis with the DNMR programme in Topsin v3.1. The hydride resonances of **Zn•Zr** were fitted over the 303 to 353 K range with an initial line broadening factor of 2 Hz. Fits for *k* were optimized to the experimental data and the modelled data are presented below. The activation parameters for the exchange of H_t with $H_{\mu'}$ are as follows: $\Delta H^{\ddagger}$ = 1.1 kJ mol^{-1}, $\Delta S^{\ddagger}$ = 0.2 J K^{-1} mol^{-1}, $\Delta G^{\ddagger}_{298K}$ = 1.0 kJ mol^{-1}. Due to the inability to observe $H_{\mu'}$ at any temperature by 1D NMR experiments (see below for EXCSY experiments), data for **Mg•Zr** have not been modelled, but a similarly low activation energy would be expected.

Figure S5. Modelled Hydride resonances of **Zn•Zr** from 293 to 363K

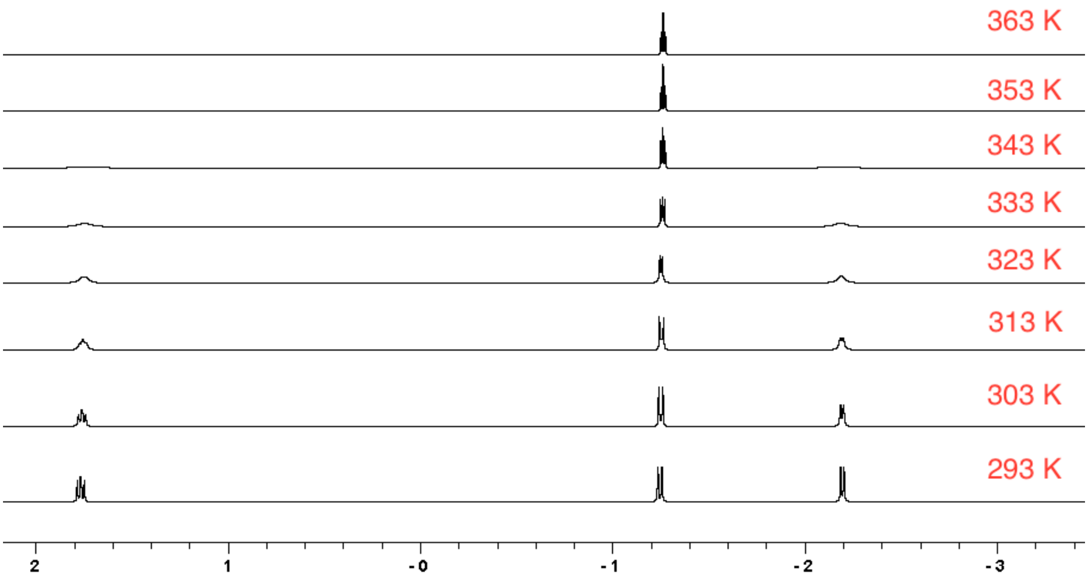

Figure S6. Eyring analysis: Ln(k/T) versus 1/T

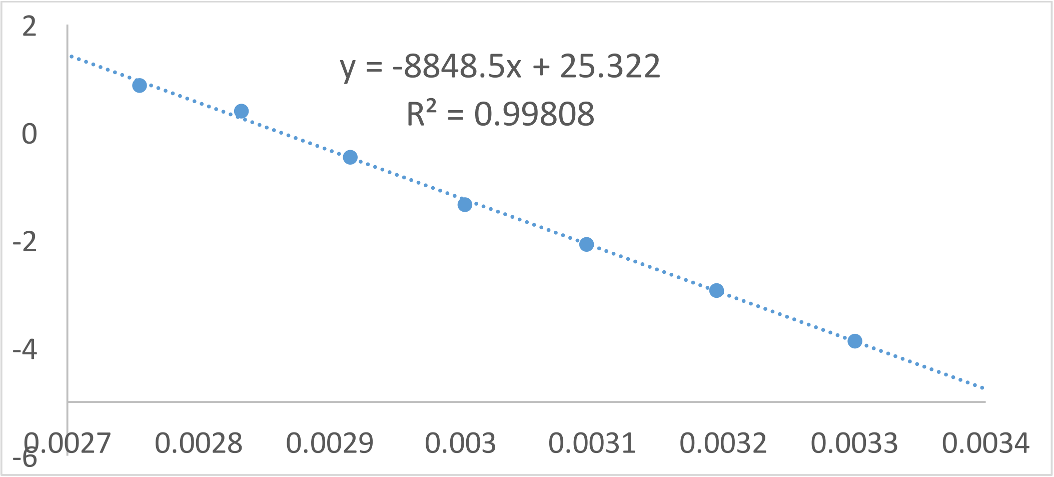

Figure S7. Irradiation at -2.02 ppm in a solution of **Mg•Zr** cooled to 273 K.

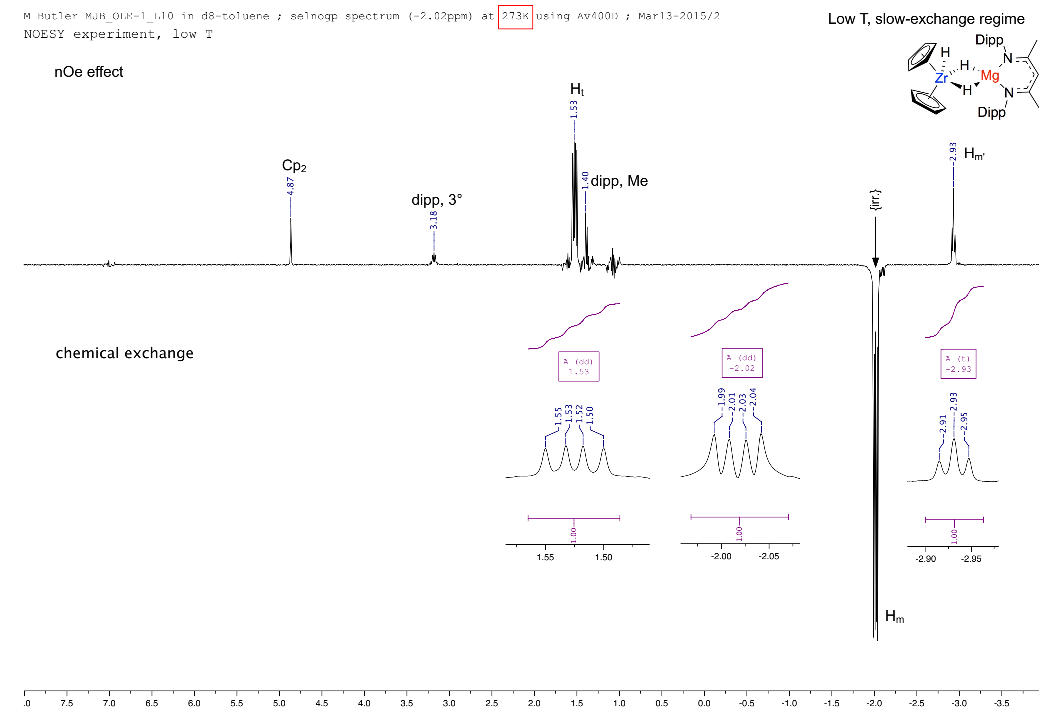

Figure S8. Irradiation at -2.93 ppm in a solution of **Mg•Zr** cooled to 273 K.

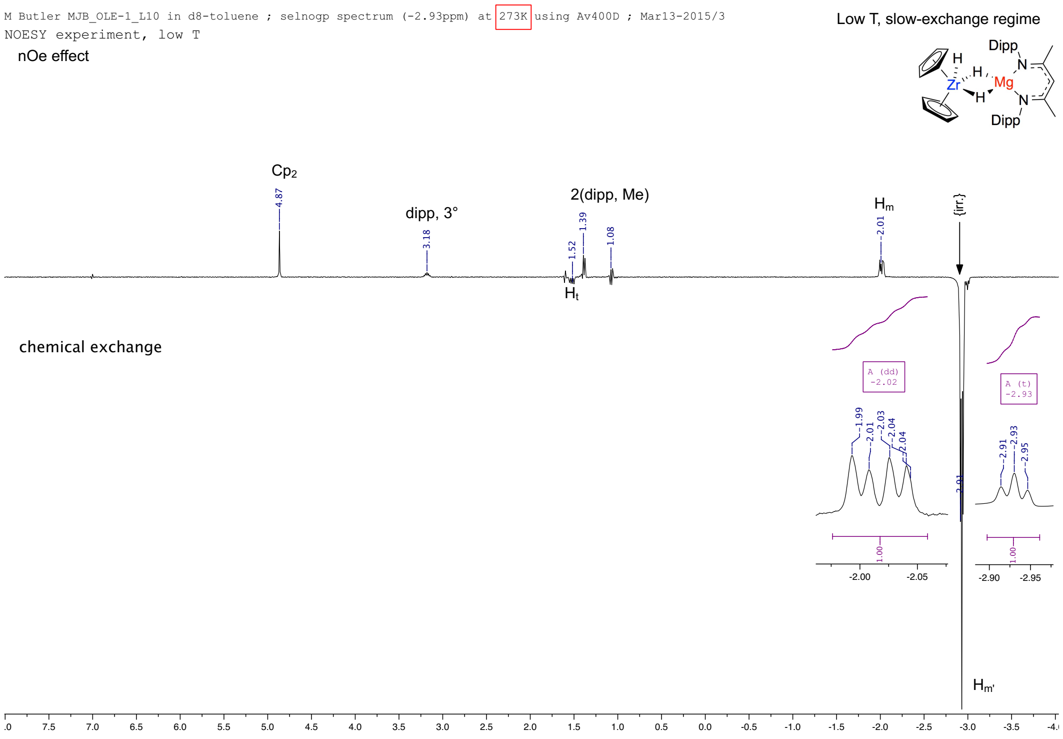

Figure S9. Irradiation at -2.05 ppm in a solution of **Mg•Zr** heated at 353 K.

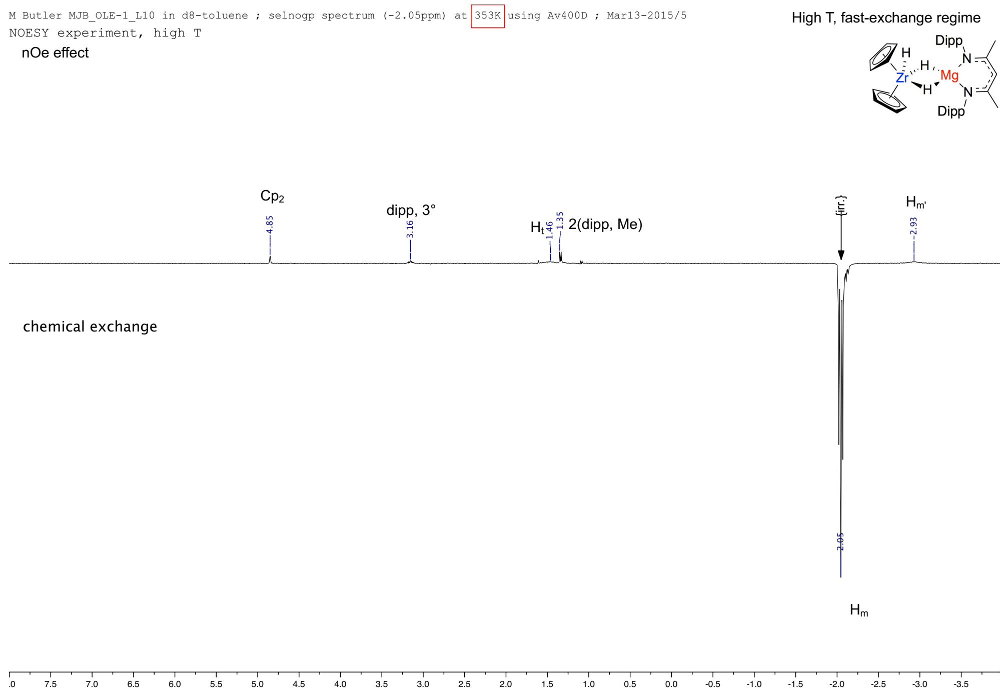

Figure S10. Irradiation at -2.93 ppm in a solution of **Mg•Zr** heated at 353 K. Observed signal enhancement for Hμ is not reproduced on irradiation of this peak (Figure S7) and may be due to signal enhancement from the original pulse.

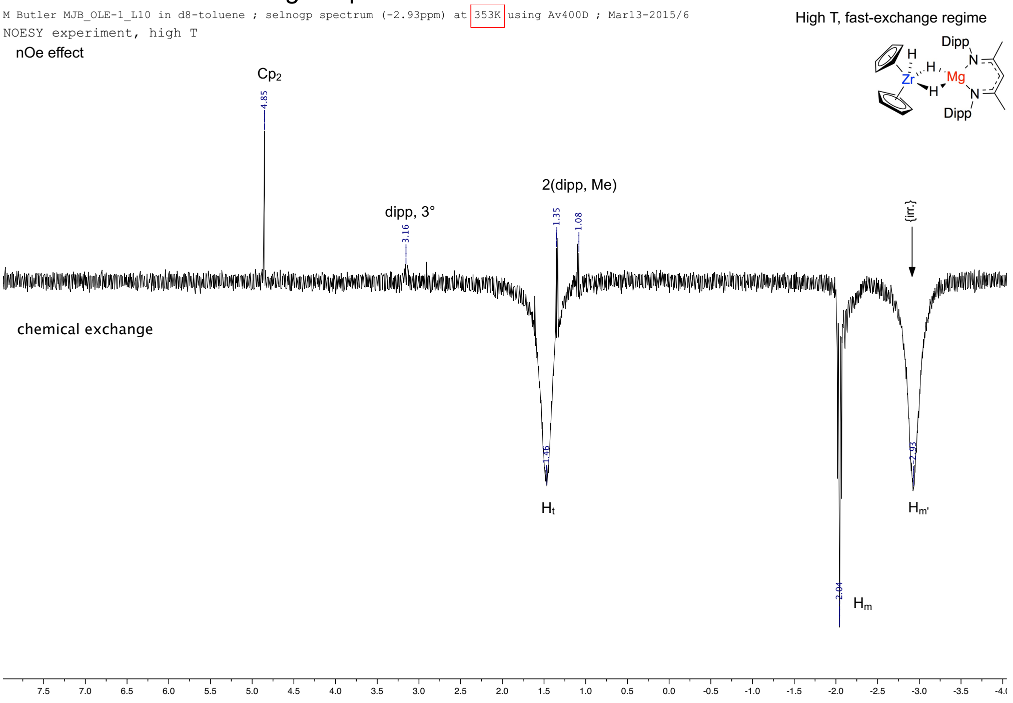

Figure S11. Irradiation at 1.78 ppm in a solution of **Zn•Zr** cooled to 273 K.

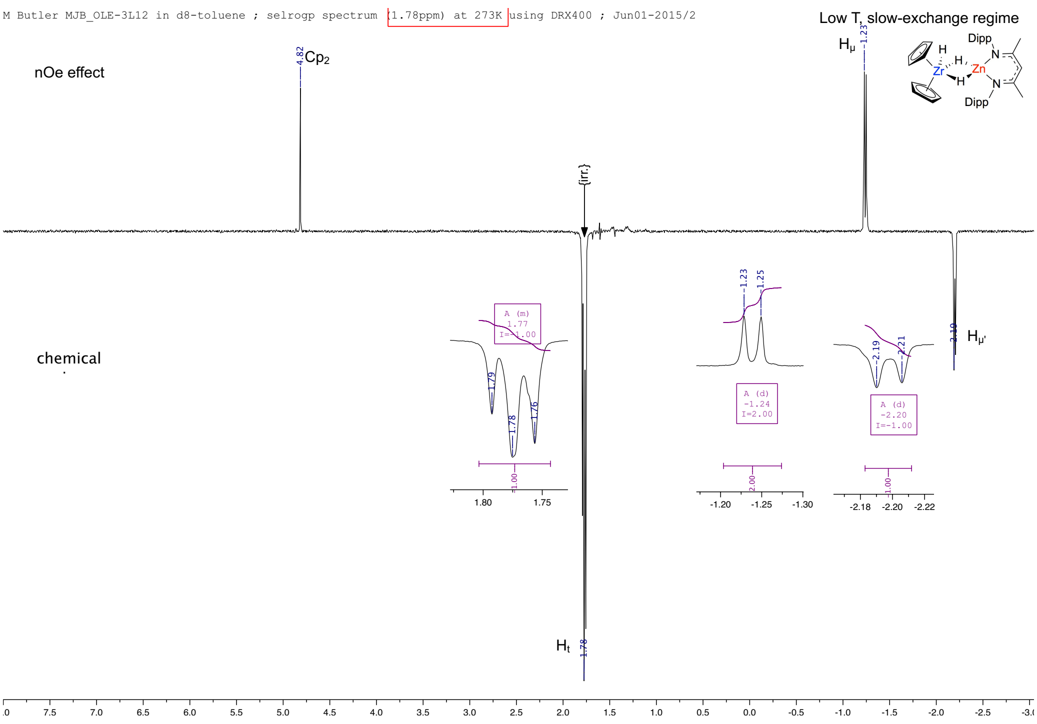

Figure S12. Irradiation at -1.24 ppm in a solution of **Zn•Zr** cooled to 273 K.

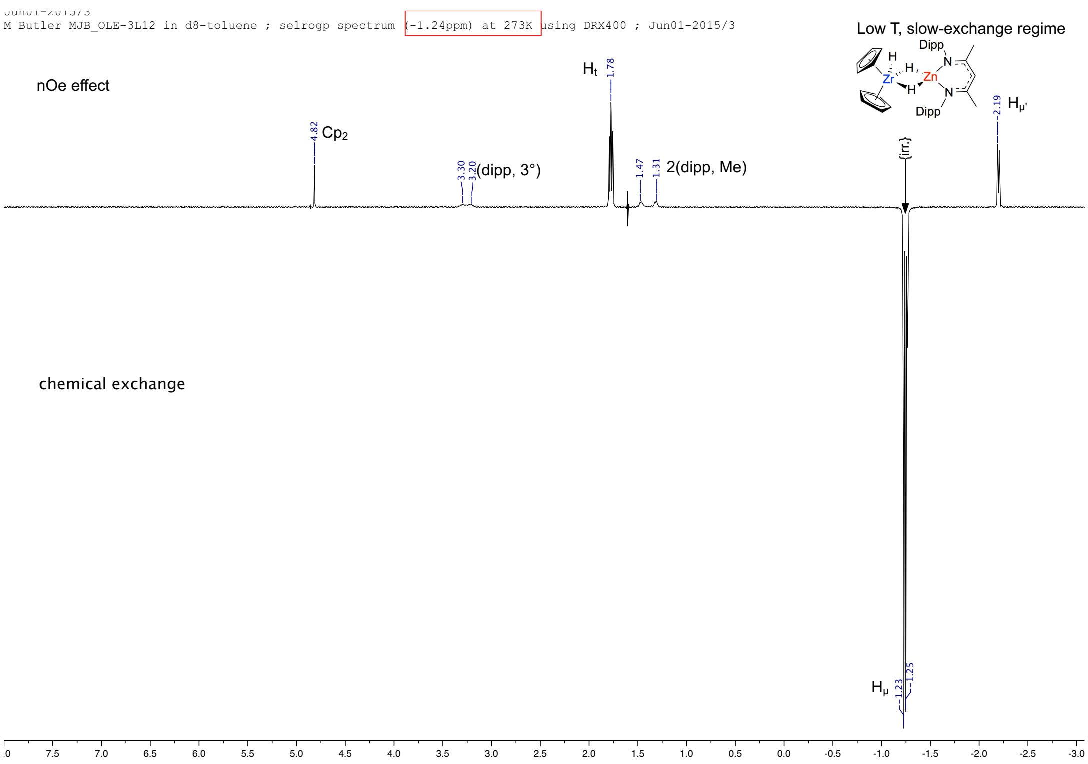

Figure S13. Irradiation at -2.20 ppm in a solution of **Zn•Zr** cooled to 273 K.

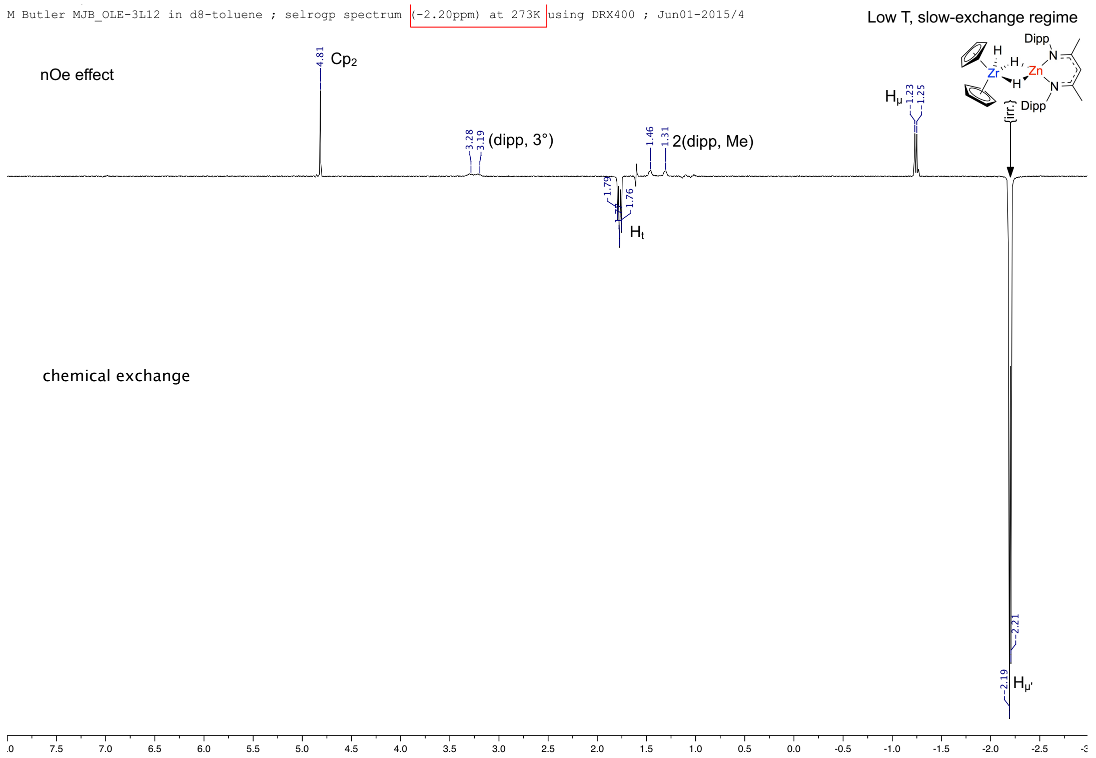

Figure S14. Irradiation at 1.74 ppm in a solution of **Zn•Zr** heated at 333 K.

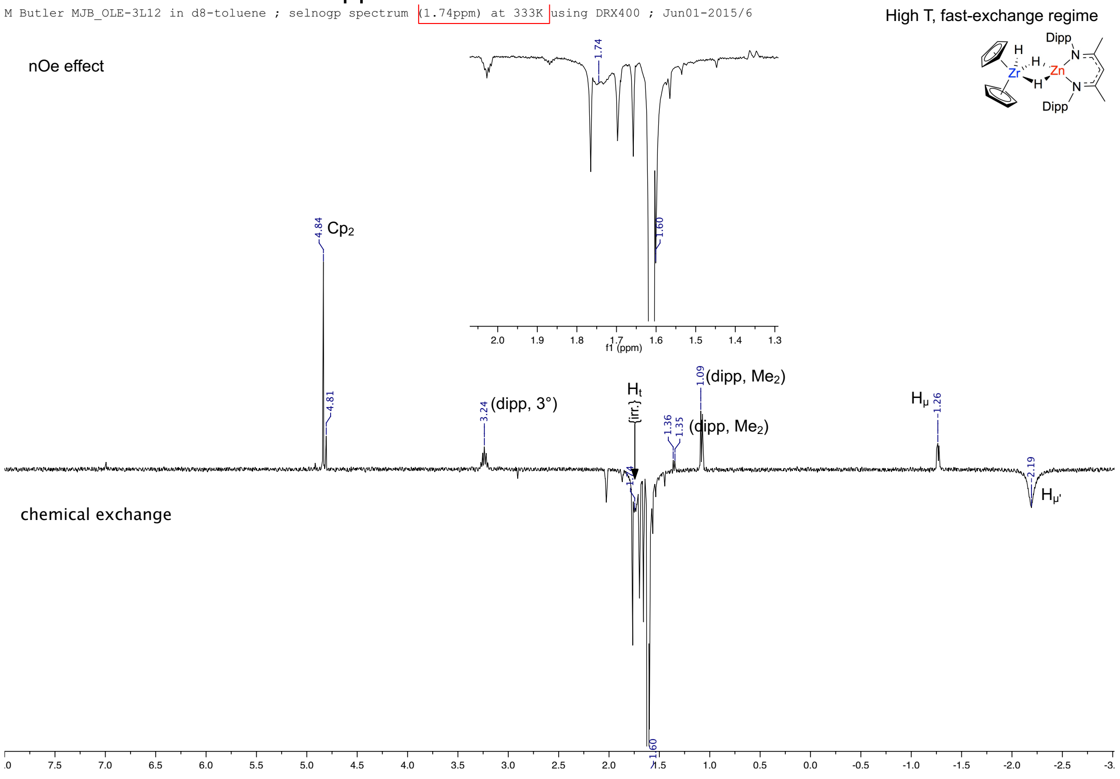

Figure S15. Irradiation at -1.26 ppm in a solution of **Zn•Zr** heated at 333 K.

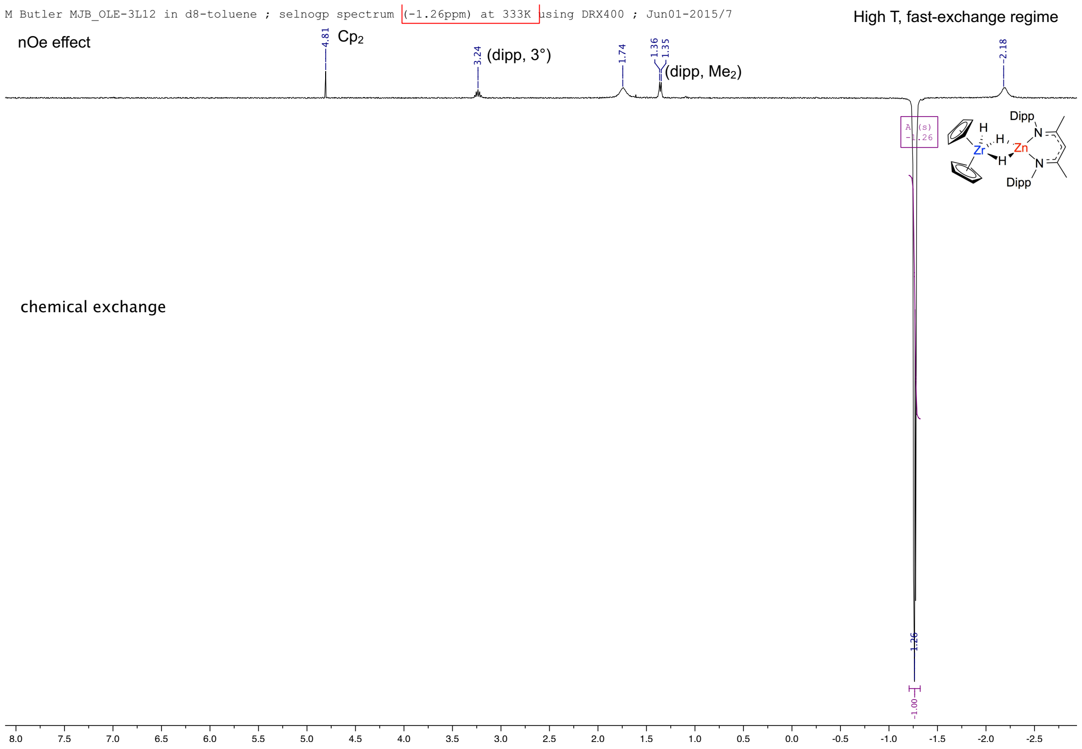

Figure S16. Irradiation at -2.19 ppm in a solution of **Zn•Zr** heated at 333 K.

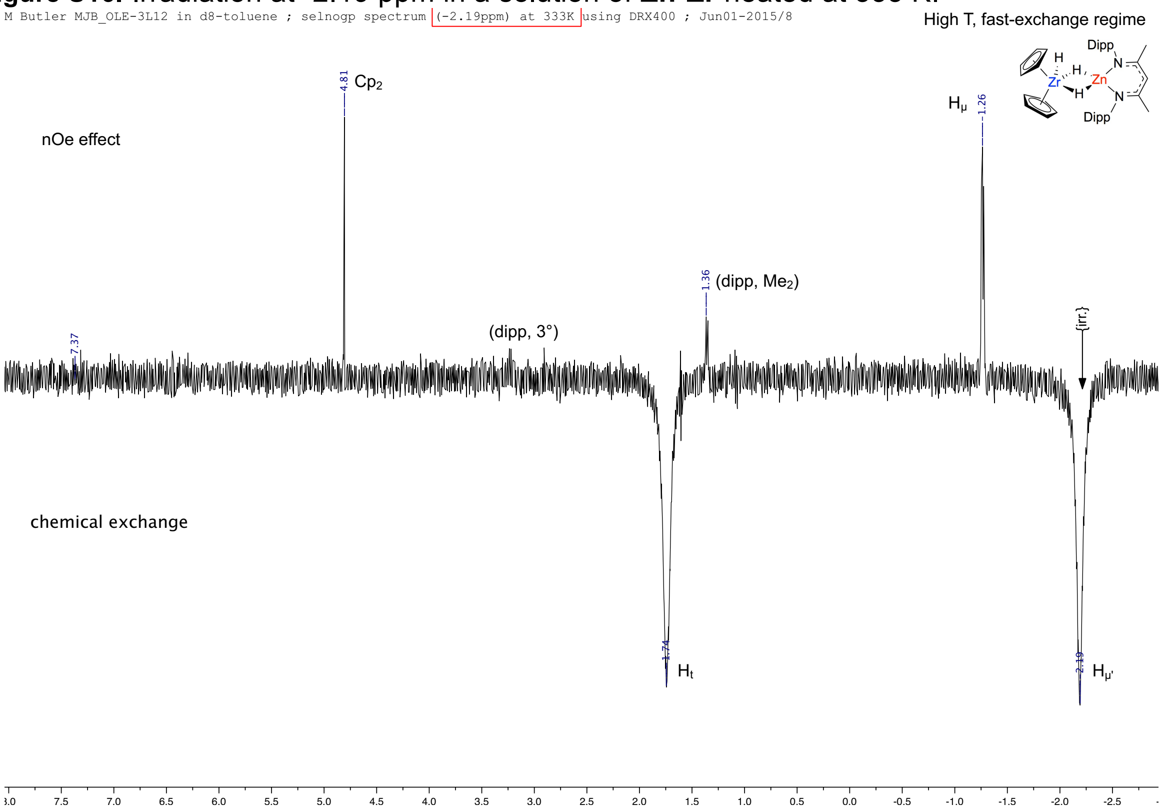

Based on the assignment of H_μ and H_μ' using DFT calculations (GIAO method) on optimized structures of **Mg•Zr** and **Zn•Zr** a mechanism can be proposed for the observed chemical exchange process between H_t and H_μ'.

Figure S17. Proposed mechanism for the chemical exchange between H_t and H_μ' in **M•Zr** (M = Zn, Mg)

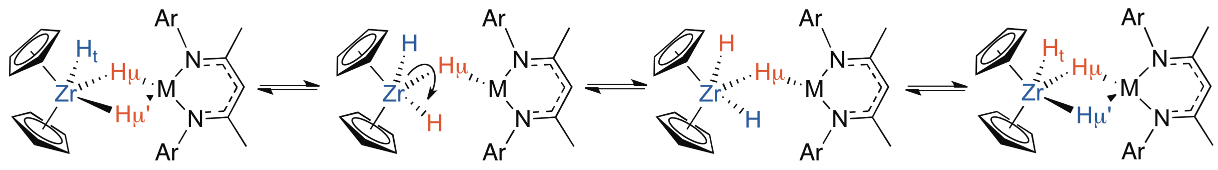

At lower temperatures, a 'freezing out' of the methyl and methine resonances assigned to the Dipp substituents occurs at 213 K in **Mg•Zr,** but at just below room temperature in **Zn•Zr**. These data are attributed to an additional fluxional process operating well below 298 K and attributed to hindered rotation around the C_{ipso}–N bond; the shorter metal---metal distance in **Zn•Zr** may contribute to the apparently higher activation energy of this processes in this analogue.

3 CATALYTIC ISOMERISATION OF CYCLOOCTA-1,3-DIENE, AND THE SYNTHESIS OF 1

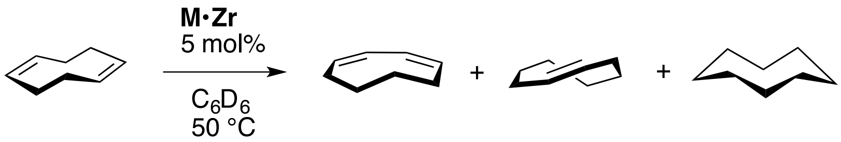

Scheme S16. Catalysis of cycloocta-1,5-COD isomerisation with **M•Zr** (M = Mg, Al, Zn and Zr) and its by-products.

Catalytic 1,5-COD isomerisation experiment - Mg•Zr and 1:

In a glovebox, 0.009 mmol of bimetallic catalyst was weighed into a 2mL scintillation vial. The solid was dissolved in 500 *µ*L C_6D_6. Roughly 2-3 mg of ferrocene was added to the vial (as internal standard for quantification). The reaction mixture was transferred to an NMR tube and cycloocta-1,5-diene (0.185 mmol, 20 equiv.) added directly by calibrated pipette. The reactions were heated to 80 °C in an oil bath. The products were confirmed by spiking product mixtures.

The combined cycloocta-1,3-diene and cyclooctene yield was calculated from ^{1}H NMR spectroscopy *in situ* (C_6D_6). Before the tube was heated to 80 °C, the substrate, cycloocta-1,5-diene, was integrated over the signal at 2.21 ppm (8H, 4x{-CH_2-}, m); the combined cycloocta-1,3-diene and cyclooctene yield was based on the integration of their conjoined peaks in the window 2.15–2.03 ppm (both multiplet peaks representing 4 H, 2x{-CH_2-}).

Catalytic 1,5-COD isomerisation experiment - Al•Zr and Zr•Zr:

In a glovebox, 500 *µ*L C_6D_6 was transferred to an NMR tube containing 2-3 mg of ferrocene (as internal standard for quantification). The cycloocta-1,5-diene (0.185 mmol, 20 equiv.) was added to the tube by calibrated pipette and a ^{1}H NMR spectrum measured. After returning the NMR tube to the glove box, the required amount of bimetallic (0.005 mmol Zr•Zr) was added to the tube. The reaction progress was monitored at room-temperature by ^{1}H NMR spectroscopy.

Table S1. Results of catalyst comparison experiments with **M•Zr** catalysts.

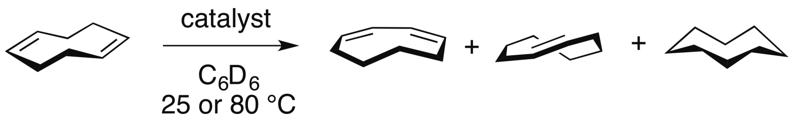

**M·Zr**	loading /mol%	temp. /°C	time /h	combined yield /%	1,3-COD :COE[a]	**M·Zr** remains /%
**Mg·Zr**	5	80	70 h	70	20:1	60
**Al·Zr**	5	25	1 h	>95[b]	11:1[b]	no
**Zr·Zr**	0.5	25	192 h	80	20:1	no
**1**	5	80	730 h	85	20:1	-

[a] The ratio is an estimate based on the relative peak heights of each product in the 2.15–2.03 ppm window. [b] Over 1 h at 25 °C, the yield diminished (240 h, 75 %) and a trace of octane was observed.

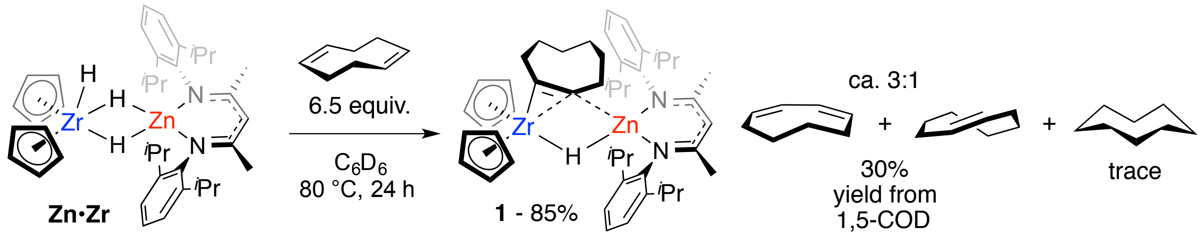

***Synthesis of 1**:* In a glovebox 84 mg of **Zn•Zr** (0.119 mmol) was weighed in a 2 mL glass scintillation vial. The material was slurried in 700 μL of C_6D_6 and 2-3 mg of ferrocene added (as internal standard for quantification). The reaction mixture was transferred by Pasteur pipette into a J Young NMR tube. 1,5-Cyclooctadiene (95 μL, 0.772 mmol, 6.5 equiv.) was then added to the reaction mixture by calibrated pipette. The NMR tube was sealed and removed from the glovebox. The reaction mixture was heated in an oil bath at 80 °C for 24 h and the course of the reaction monitored by ^{1}H NMR spectroscopy (giving an 85% yield of **1** by internal standard). The NMR tube was returned to the glovebox, and the mixture was transferred to a 20 mL scintillation vial. The solution was concentrated *in vacuo*, leaving a yellow solid. The crude material was triturated with 2 mL of *n*-hexane; the resulting yellow supernatant liquid was decanted and the remaining material recrystallised from toluene at -30 °C. The colourless crystalline solid **1** was collected as three crops (24.6 mg, 0.296 mmol, 25%).

^{1}H NMR data (C_6D_6, 298K, 500 MHz): δ -1.22 (s, 1H, Zr*H*), 1.12 (d, 6H, *J* = 6.5 Hz, CH*Me*$_2$), 1.25 (d, 12H, *J* = 6.5 Hz, C*H*Me$_2$), 1.43-1.47 (m, 2H), 1.62 (d, 6H, *J* = 6.5 Hz), 1.72 (s, 6H, *Me*), 1.70-1.76 (m, 2H), 1.92-1.98 (m, 2H), 3.15-3.18 (m, 4H), 3.21 (heptet, 2H, *J* = 6.5 Hz), 3.79 (heptet, 2H, *J* = 6.5 Hz), 4.87 (s, 1H, C*H*), 4.91 (s, 10H, Cp*H*), 7.06-7.13 (m, 4H, Ar*H*), 7.22 (d, 2H, *J* = 7.5 Hz). ^{13}C NMR data (C_6D_6, 298K, 100 MHz): δ 24.3 (CH*Me*$_2$), 24.4 (CH*Me*$_2$), 24.8 (*Me*), 25.3 (CH*Me*$_2$), 25.5 (CH*Me*$_2$), 25.6 (*C*H$_2$), 27.5 (*C*H$_2$), 27.6 (*C*H$_2$), 28.3 (*C*HMe$_2$), 29.0 (*C*HMe$_2$), 29.2 (*C*H$_2$), 34.0 (*C*H$_2$), 37.0 (*C*H$_2$), 95.2 (*C*H), 102.6 (*Cp*), 111.9 (*C*≡*C*), 124.2 (*Ar*H), 124.6 (*Ar*H), 126.1 (*Ar*H), 142.6 (quart. *Ar*), 143.0 (quart. *Ar*), 147.0 (quart. *Ar*), 167.4 (*C*=N), 184.3 (*C*≡*C*). Infrared (solid, cm^{-1}) 3128 (w), 3064 (w), 2960 (m), 2918 (m), 2868 (m), 1612 (w), 1547 (m), 1519 (m), 1432 (s), 1404 (s), 1360 (m), 1311 (s), 1229 (m), 1175 (m), 1097 (m), 1059 (w), 1021 (m). Repeated attempts to acquire satisfactory CHN analysis failed due to the air and moisture sensitive nature of this complex.

Figure S18. VT NMR data on **1** 193 to 363 K in toluene-d_8: VT NMR data in toluene-d_8 revealed that **1** retains its integrity in solution, undergoing little or no change across the 193 to 353 K temperature range.

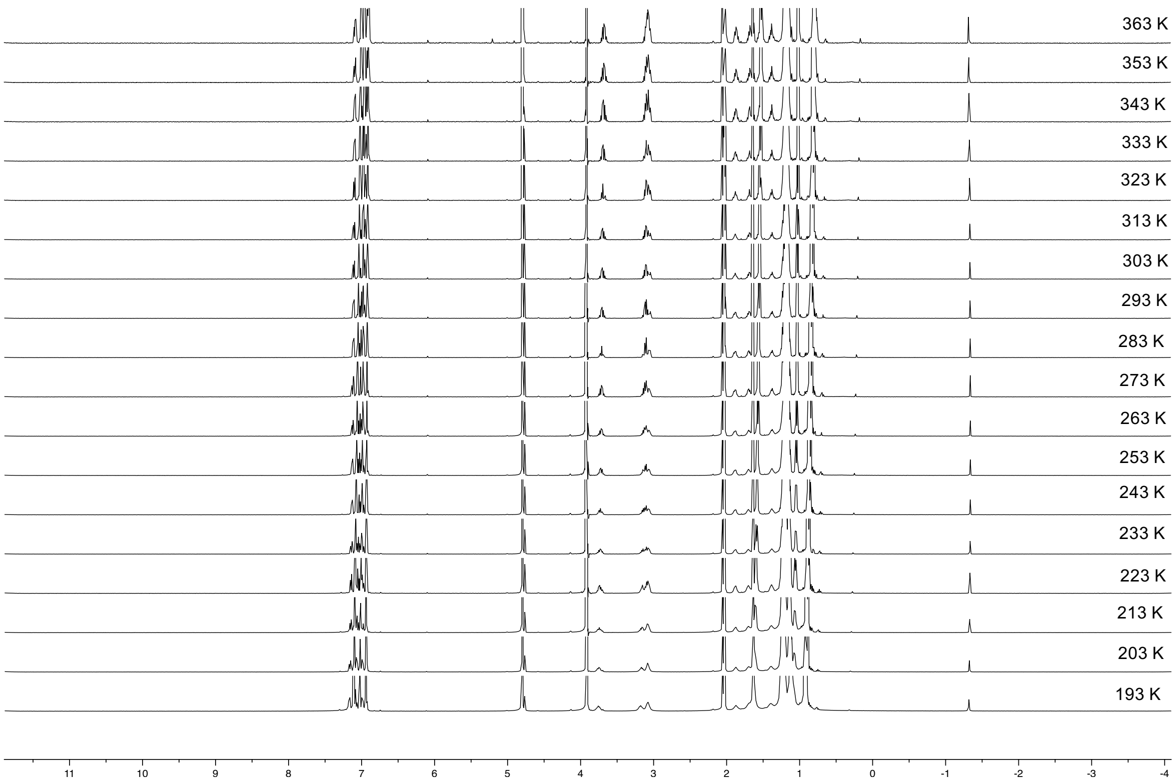

Figure S19. NOE spectrum on **1** in toluene-d_8

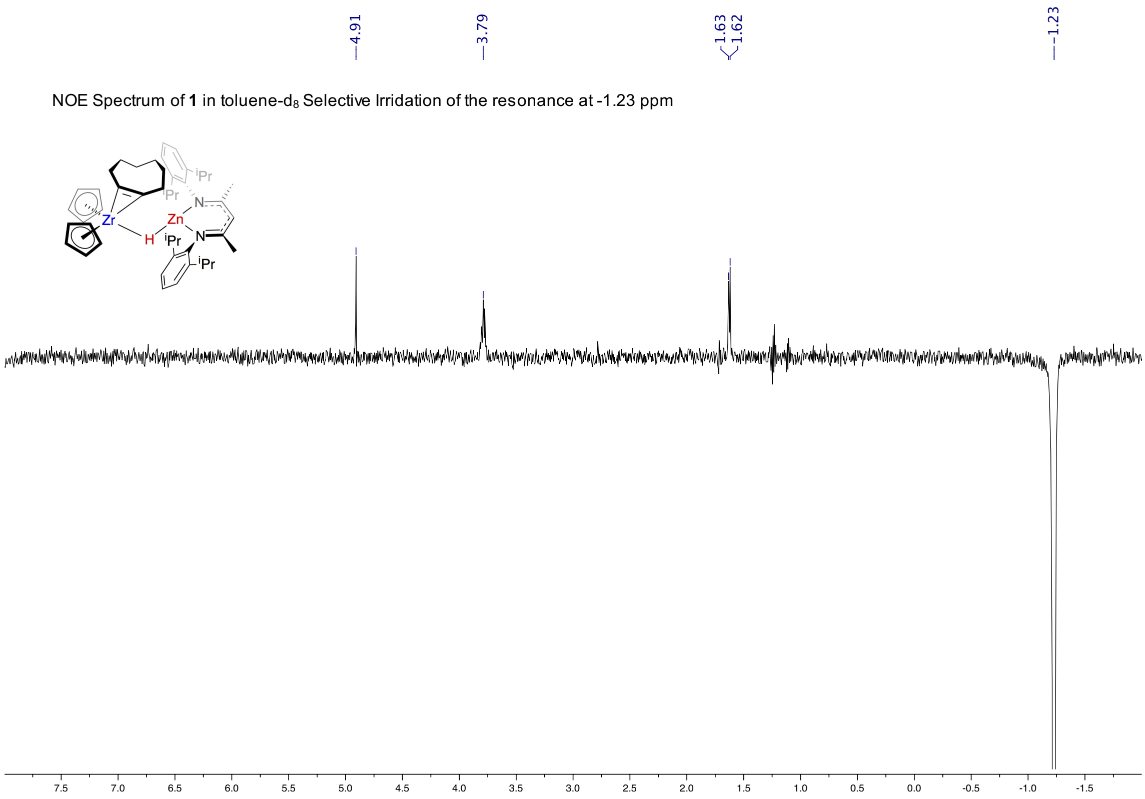

4 DFT STUDIES

Calculations were conducted in Gaussian09. All minima were confirmed by frequency calculations and solid-state data were used as an input for the atom coordinates. NBO calculations were run using NBO v5.9 within g09. A series of functionals (B3LYP, M-062x, ωB97x, ωB97xD) and basis-sets (6,311G+(d,p) / 6,31G+(d,p) / LanL2DZ / SDD) were investigated. Bader analysis was conducted on optimized geometries in the AIMALL package. In all cases the geometries were compared against the solid-state data. For **M•Zr** an m062x functional and hybrid basis set 6,31G+(d,p) (C/H/N) and Lanl2DZ (Al/Zn/Mg/Zr) was employed. For **1**, benchmarking experiments demonstrated that the ωB97x functional and hybrid basis-set 6,311G+(d,p) (C/H/N) and SDD (Zn/Zr) better reproduce the solid state data and specifically the intermetallic distance and Zn–atom distances. The relative energies of isomers of C_8H_{12} were calculated using the B3LYP functional and 6,311G+(d,p) basis set, conformation searches were performed manually by initiating calculations from a large number of conformations in g09.[5]

Figure S20. Relative stability of isomers of C_8H_{12}

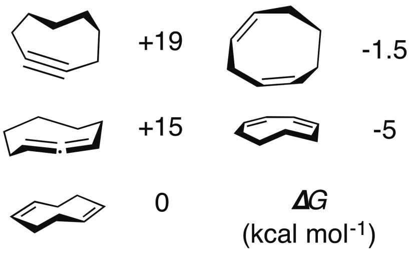

[5] The relative stabilities of 1,3-COD, 1,4-COD and 1,5-COD are consistent with experimental thermochemical data from E. Taskinen and K. Nummelin, *Acta. Chemica, Scandinavica* B 39, **1985**, 791.

Table S2. Calculated bond angles and Bond lengths in **M•Zr** (M = Zn, Mg, Al)

	**M = Zn**	**M = Mg**	**M = Al**
**Zr–H$_b$**	1.98,1.99	1.96,1.98	1.95,1.94
**Zr–H$_t$**	1.82	1.8	1.83
**Zr---M**	3.02	3.10	3.06
**M–H$_b$**	1.82,1.85	1.88, 1.90	1.77, 1.90
**H$_b$–Zr–H$_{b'}$**	72.2	71.3	68.3

Table S3. Wiberg Bond Indices in **M•Zr.** 6,31G+(d,p) (C/H/N) and Lanl2DZ (Al/Zn/Mg/Zr)

	Zr---M	Zr–H$_t$	Zr–H$_b$	M–H$_b$
**M = Zn**	0.08	0.80	0.59,0.60	0.13,0.13
**M = Mg**	0.04	0.80	0.61,0.61	0.09,0.10
**M = Al**	0.15	0.81	0.47,0.55	0.27,0.34

a – WBI of the terminal Al–H is 0.75

Table S4: Natural Population Analysis – Charge

	Zr	M	H$_t$	H$_b$
**M = Zn**	0.83	1.63	-0.19	-0.38,-0.42
**M = Mg**	0.86	1.73	-0.19	-0.41,-0.44
**M = Al**	0.93	1.53	-0.20	-0.31,-0.31

a – NPA charge on the terminal Al–H is –0.43

Figure S21: Optimized structures (DFT, gas phase calculations) of (a) **Mg•Zr**, (b) **Zn•Zr** and **(c) Al•Zr**

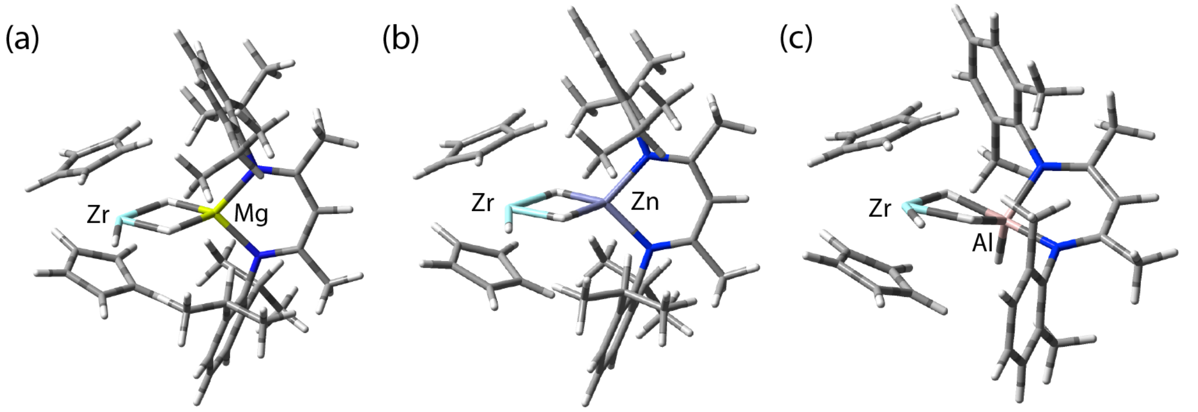

Figure S22. Comparison of experimental bond angles and bond lengths of the Zr---Zn core of molecule B within the unit cell of **1** against calculated values using a truncated model in which *i*-Pr groups have been replaced with methyl groups.

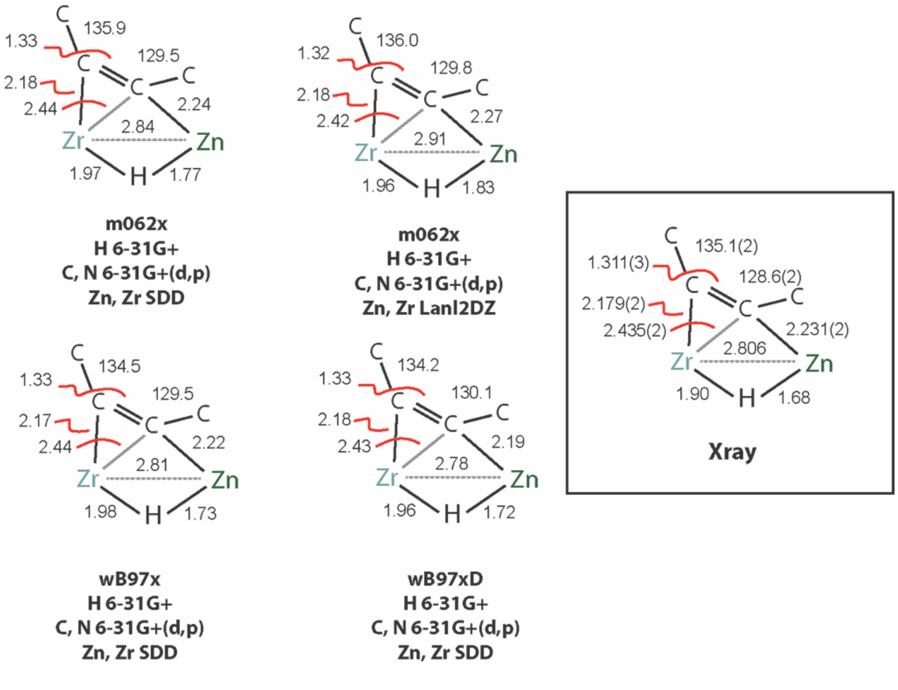

Figure S23. Comparison of calculated parameters of **1** using molecule **A** and **B** from the X-ray crystallographic data as inputs. No truncations made.

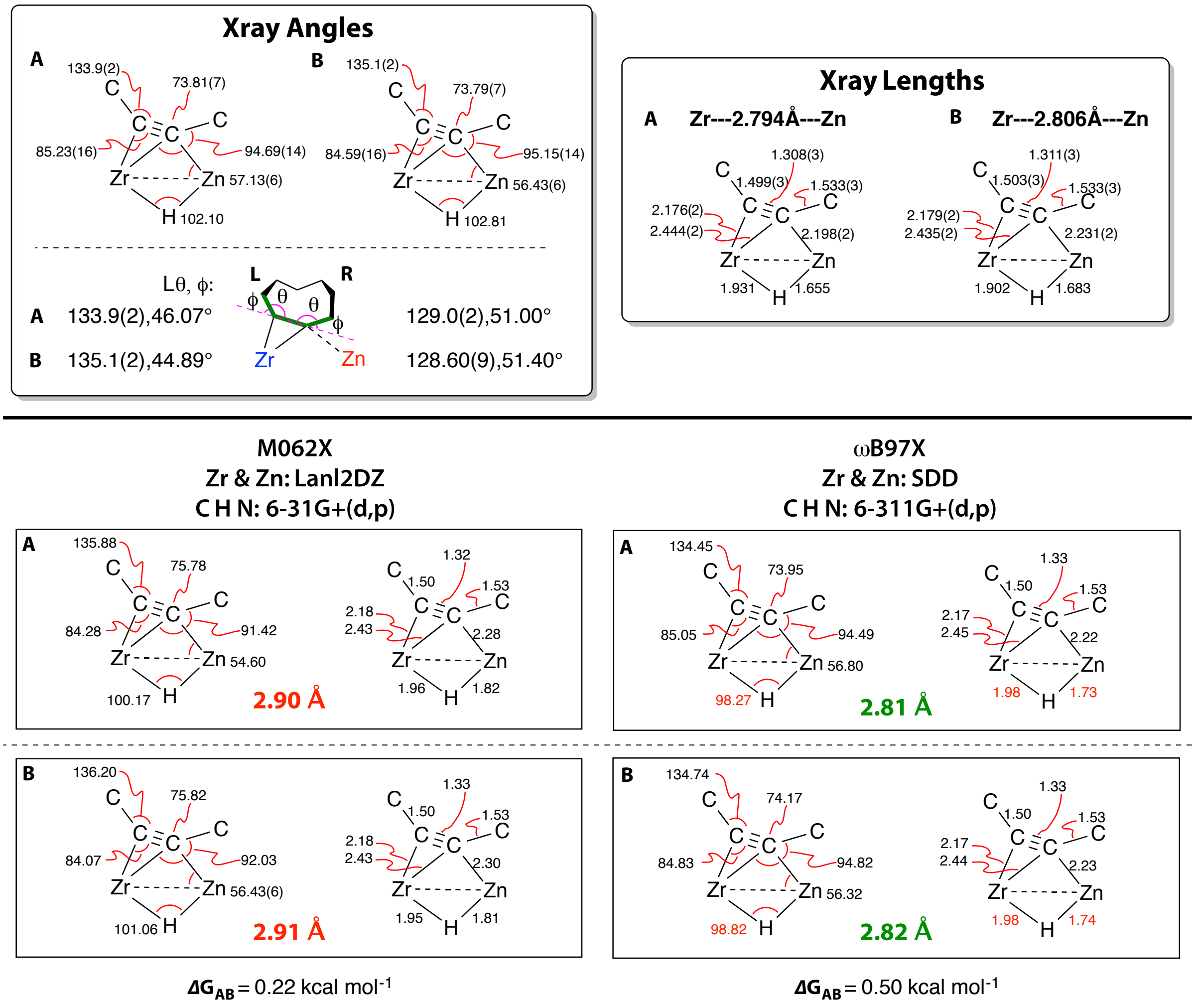

Figure S24. Fragment analysis using molecule B in the unit cell: electronic energies (E_0) were used to calculate all ΔE values. The difference in E_0 between the single point calculations on fragments give $\Delta E_{0,dist1}$ (heterobimetallic unit) and $\Delta E_{0,dist2}$ (cyclooctyne unit). ωB97x functional and hybrid basis-set 6,311G+(d,p) (C/H/N) and SDD (Zn/Zr)

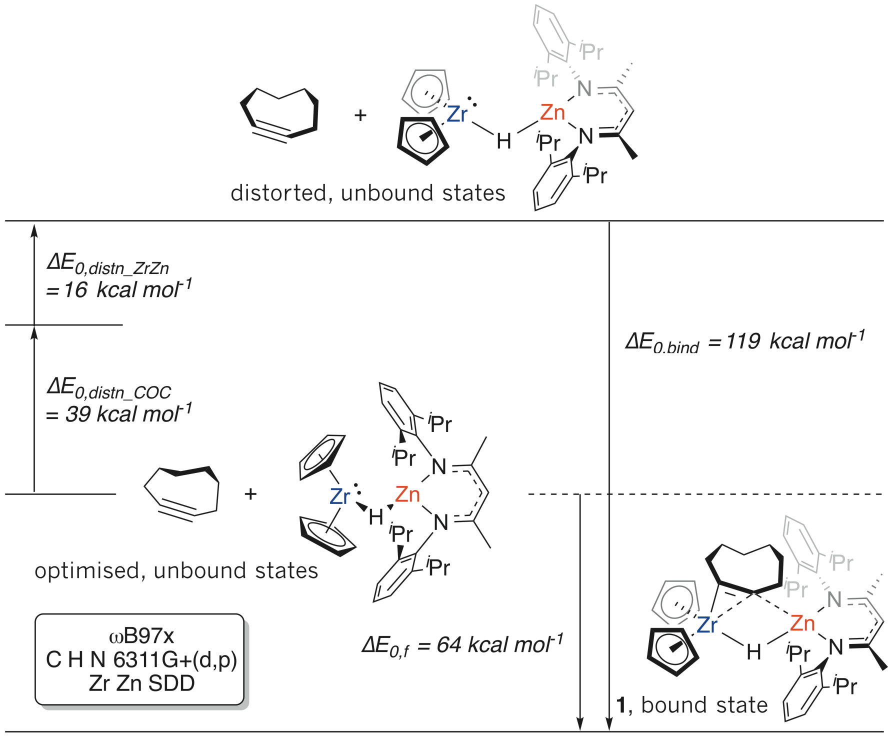

Figure S25. Fragment analysis using molecule B in the unit cell: electronic energies (E_0) were used to calculate all ΔE values. The difference in E_0 between the single point calculations on fragments give $\Delta E_{0,dist\_Zn}$ (Zn unit) and $\Delta E_{0,distn\_Zr}$ (Zr unit). ωB97x functional and hybrid basis-set 6,311G+(d,p) (C/H/N) and SDD (Zn/Zr)

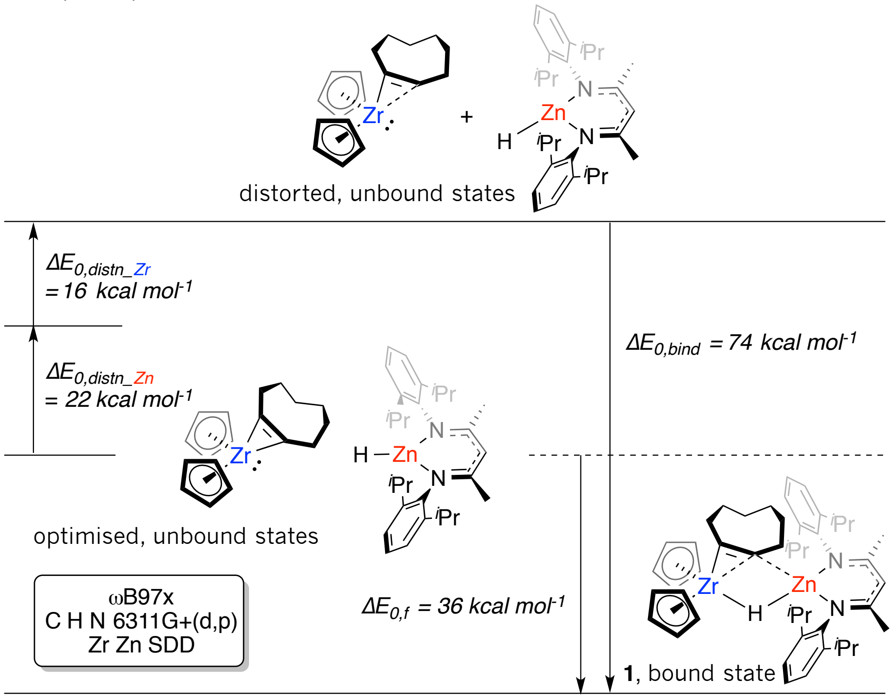

Figure S26. Fragment analysis using molecule B in the unit cell: electronic energies (E_0) were used to calculate all ΔE values. The difference in E_0 between the single point calculations on fragments give $\Delta E_{0,distn\_Zr}$ (Zn$^-$ unit) and $\Delta E_{0,distn\_Zn}$ (Zn$^+$ unit). ωB97x functional and hybrid basis-set 6,311G+(d,p) (C/H/N) and SDD (Zn/Zr)

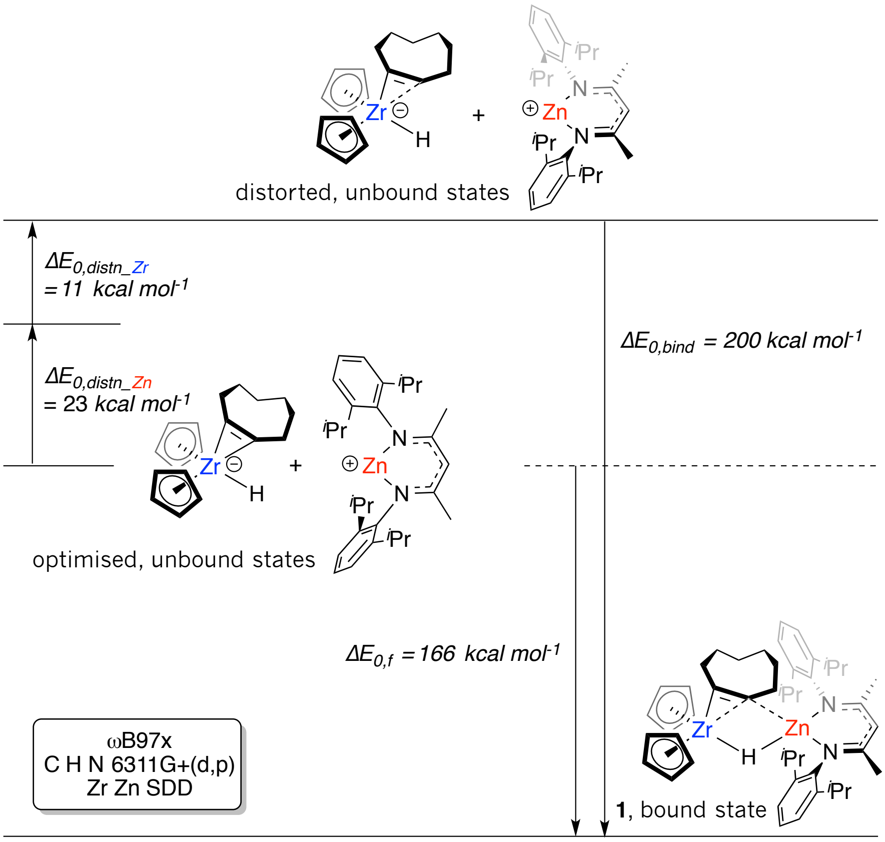

Table S5. NBO analysis. NPA charges and Wiberg Bond Indices of **1**. ωB97x functional and hybrid basis-set 6,311G+(d,p) (C/H/N) and SDD (Zn/Zr)

NPA Charges			Wiberg Bond Indices
Zr	+0.97	Zr---Zn	0.10
Zn	+1.59	Zn–C_β	0.13
C_α	-0.29	Zr–C_α	0.58
C_β	-0.44	Zr–C_β	0.76
H–(Zr)	-0.39	Zr–H	0.60
		Zn–H	0.16
		C_α–C_β	1.87

5 X-RAY DATA

Crystal structures were obtained from the (colourless) single crystals of **M•Zr** grown from hexane at -20 °C. Both of the crystals gave a unit cell containing two very slightly different heterobimetallic structures, whose vital dimensions are within error. Both X-ray diffraction studies that reveal roughly flat over-all shapes (ignoring the Dipp substituents), with intermetallic areas that are less sterically crowded on one face. This opening is most likely to be the location of the H_t. Notably, the cleft seems to arise from a slight (ca. 18.3°) twisting of one Cp centroid away from the (Nacnac)Mg plane in **Mg•Zr**. This is not the case **Zn•Zr**, whose (Nacnac)Zn metallacycle adopts a shallow boat structure with Zn and C_β bow and stern. The eclipsed Cp ligands and Dipp groups drop with the hull, leaving the open region above the co-planar N,N-Zn---Zr.

The X-ray crystal structure of Mg·Zr

Crystal data for **Mg·Zr**: $C_{39}H_{54}MgN_2Zr{\cdot}C_7H_8$, M = 758.50, triclinic, P-1 (no. 2), a = 11.03001(18), b = 18.1450(3), c = 22.1419(8) Å, α = 106.340(3), β = 90.840(3), γ = 90.0603(13)°, V = 4251.96(18) Å^3, Z = 4 (two independent molecules), ρ_{calcd} = 1.185 g cm^{-3}, $\mu(Mo_{K\alpha})$ = 0.305 mm^{-1}, T = 173 K, yellow blocks, Agilent Xcalibur 3 E diffractometer; 19516 independent measured reflections, F^2 refinement,[6] R_1(obs) = 0.1080, wR_2(all) = 0.3178, 13970 independent observed absorption-corrected reflections [$|F_o| > 4\sigma(|F_o|)$, $2\theta_{max}$ = 58°], 937 parameters. CCDC 1454102.

The crystal of **Mg·Zr** that was studied was found to be severely twinned. The best results were obtained from a three component twin model in a *ca*. 61:33:6 ratio (though at least one more component could be seen in the data), with the two major lattices related by the approximate twin law [–1.00 0.00 0.00 0.00 1.00 0.00 0.00 –0.05 –1.00]. The structure was found to contain two independent complexes, **Mg·Zr-A** and **Mg·Zr-B**, and the presumed three Zr–H and Zr–H–Mg hydrogen atoms could not be located, so the atom list for the

[6] (a) SHELXTL, Bruker AXS, Madison, WI; (b) SHELX-97, G.M. Sheldrick, *Acta Cryst.*, **2008**, *A64*, 112-122; (c) SHELX-2013, http://shelx.uni-ac.gwdg.de/SHELX/index.php

asymmetric unit is low by 6 hydrogen atoms, and that for the unit cell is low by 12 hydrogen atoms. The C40- and C50-based included toluene solvent molecules were both found to be disordered, and in each case two orientations were identified, of *ca*. 74:26 and 58:42% occupancy respectively. The geometries of all four orientations were optimised, the thermal parameters of adjacent atoms were restrained to be similar, and only the non-hydrogen atoms of the major occupancy orientations were refined anisotropically (those of the minor occupancy orientations were refined isotropically).

The X-ray crystal structure of Zn·Zr

Crystal data for **Zn·Zr**: $C_{39}H_{54}N_2ZnZr$, M = 707.43, monoclinic, $P2_1/m$ (no. 11), a = 9.0201(4), b = 20.8878(7), c = 19.4369(6) Å, β = 91.350(3)°, V = 3661.1(2) Å^3, Z = 4 (two independent C_S-symmetric molecules), ρ_{calcd} = 1.283 g cm^{-3}, μ($Mo_{K\alpha}$) = 0.967 mm^{-1}, T = 173 K, colourless tablets, Agilent Xcalibur 3 E diffractometer; 7496 independent measured reflections (R_{int} = 0.0286), F^2 refinement, R_1(obs) = 0.0537, wR_2(all) = 0.1319, 6245 independent observed absorption-corrected reflections [$|F_o| > 4\sigma(|F_o|)$, $2\theta_{max}$ = 57°], 408 parameters. CCDC 1454103.

The structure of **Zn·Zr** was found to contain two independent complexes, **Zn·Zr-A** and **Zn·Zr-B**, both of which sit across mirror planes that pass through the zirconium and zinc centres and bisect the diketimine ligand. The presumed three Zr–H and Zr–H–Zn hydrogen atoms could not be located, so the atom list for the asymmetric unit is low by 3 hydrogen atoms, and that for the unit cell is low by 12 hydrogen atoms.

The X-ray crystal structure of 1

Crystal data for **1**: $C_{47}H_{64}N_2ZnZr$, M = 813.59, monoclinic, $P2_1/c$ (no. 14), a = 16.4692(5), b = 36.5002(10), c = 14.0481(4) Å, β = 103.342(3)°, V = 8216.8(4) Å^3, Z = 8 (two independent molecules), ρ_{calcd} = 1.315 g cm^{-3}, μ($Mo_{K\alpha}$) = 0.871 mm^{-1}, T = 173 K, colourless tablets, Agilent Xcalibur 3 E diffractometer; 16152 independent measured reflections (R_{int} = 0.0204), F^2 refinement,[7] R_1(obs) = 0.0364, wR_2(all) = 0.0919, 12707 independent observed

---

[7] (a) SHELXTL, Bruker AXS, Madison, WI; (b) SHELX-97, G.M. Sheldrick, *Acta Cryst.*, **2008**, *A64*, 112-122; (c) SHELX-2013, http://shelx.uni-ac.gwdg.de/SHELX/index.php

absorption-corrected reflections [$|F_o| > 4\sigma(|F_o|)$, $2\theta_{max} = 57°$], 955 parameters. CCDC 1454104.

The structure of **1** was found to contain two independent complexes, **1-A** and **1-B**. For each complex the Zr–H–Zn bridging hydrogen atom was located from a Δ*F* map and refined freely. The standard calculated position for the C2–H hydrogen atom in each complex was found to differ significantly from the observed electron density in the Δ*F* map, and so these hydrogen atoms were placed in the found positions and then refined freely subject to a C–H distance constraint of 0.950 Å (the distance that would have been used in the standard treatment).

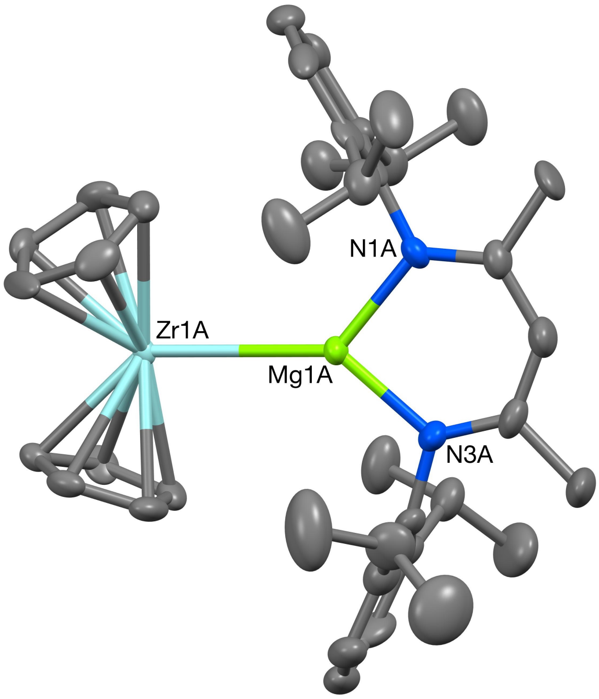

Figure S27. The structure of one (**Mg·Zr-A**) of the two independent complexes present in the crystal of **Mg·Zr** (50% probability ellipsoids).

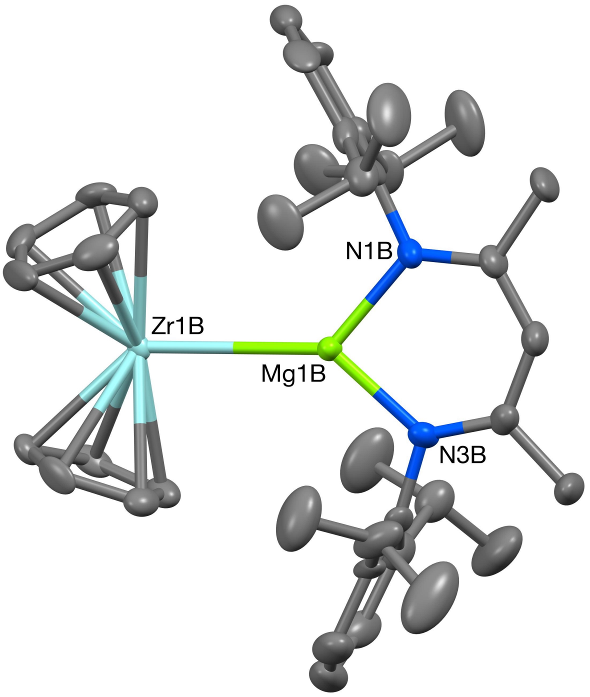

Figure S28. The structure of one (**Mg·Zr-B**) of the two independent complexes present in the crystal of **Mg·Zr** (50% probability ellipsoids).

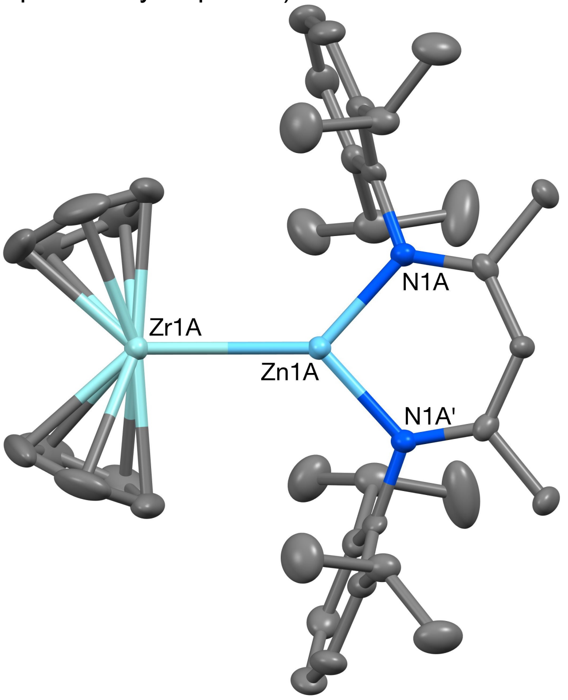

Figure S29 The structure of one (**Zn·Zr-A**) of the two independent C_S-symmetric complexes present in the crystal of **Zn·Zr** (50% probability ellipsoids).

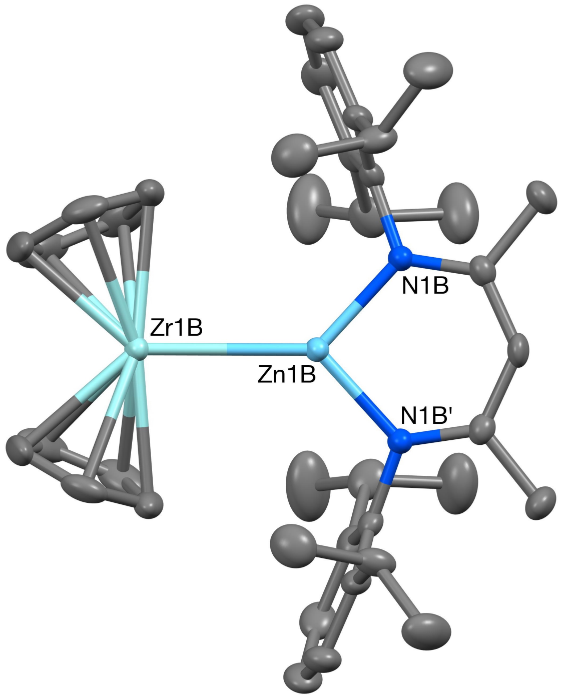

Figure S30 The structure of one (**Zn·Zr-B**) of the two independent C_S-symmetric complexes present in the crystal of **Zn·Zr** (50% probability ellipsoids).

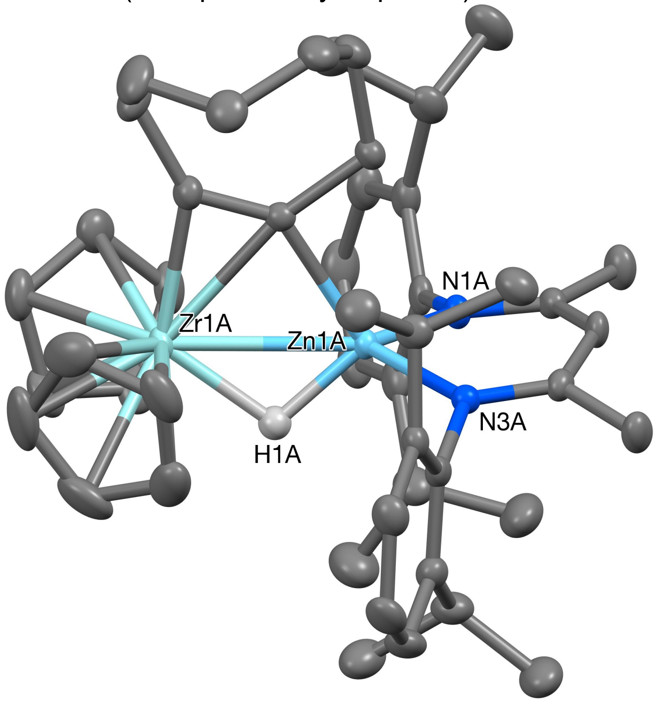

Figure S31. The structure of one (**1-A**) of the two independent complexes present in the crystal of **1** (50% probability ellipsoids).

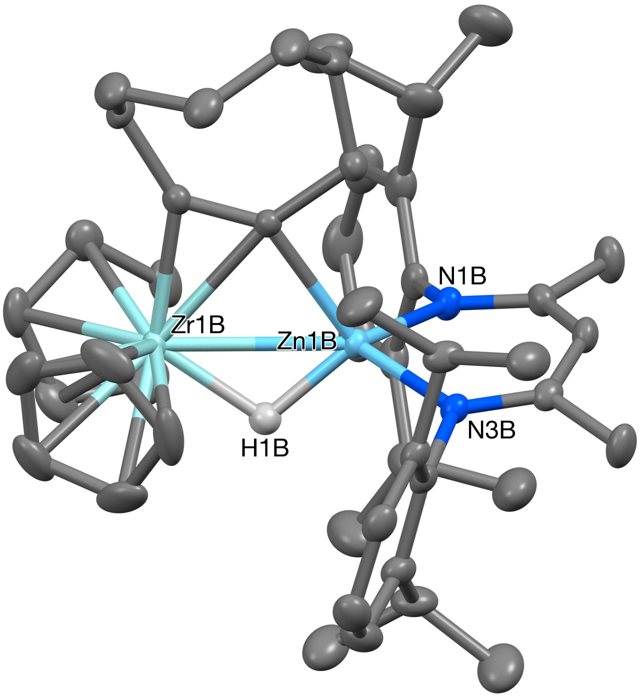

Figure S32. The structure of one (**1-B**) of the two independent complexes present in the crystal of **1** (50% probability ellipsoids).

6 MULTINUCLEAR NMR DATA

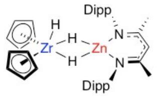

^{1}H NMR (C_6D_6, 298 K, 400 MHz)

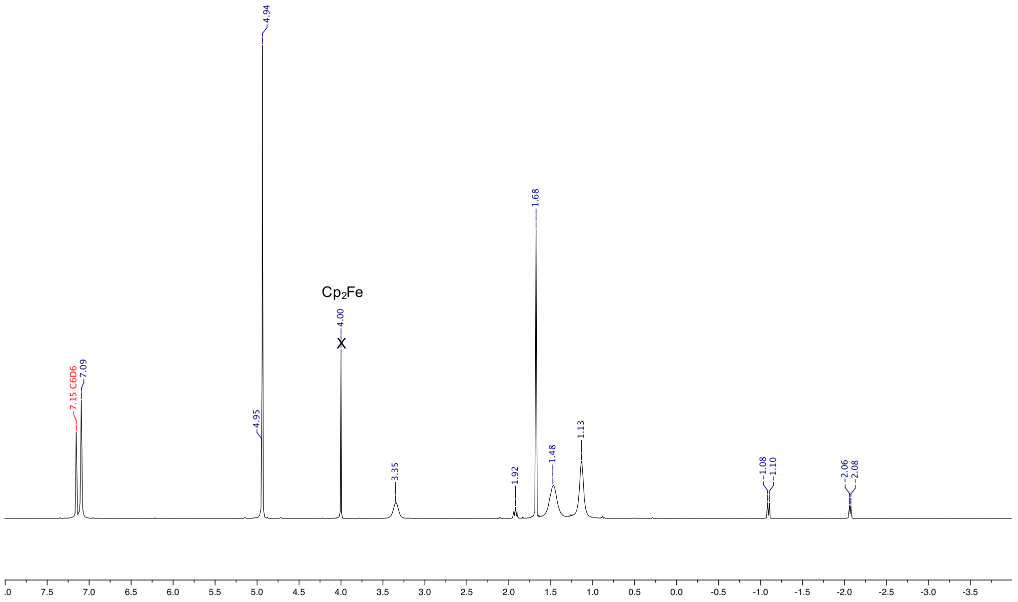

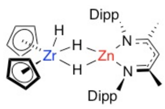

$^{13}C\{^{1}H\}$ NMR (C_6D_6, 298 K, 100 MHz)

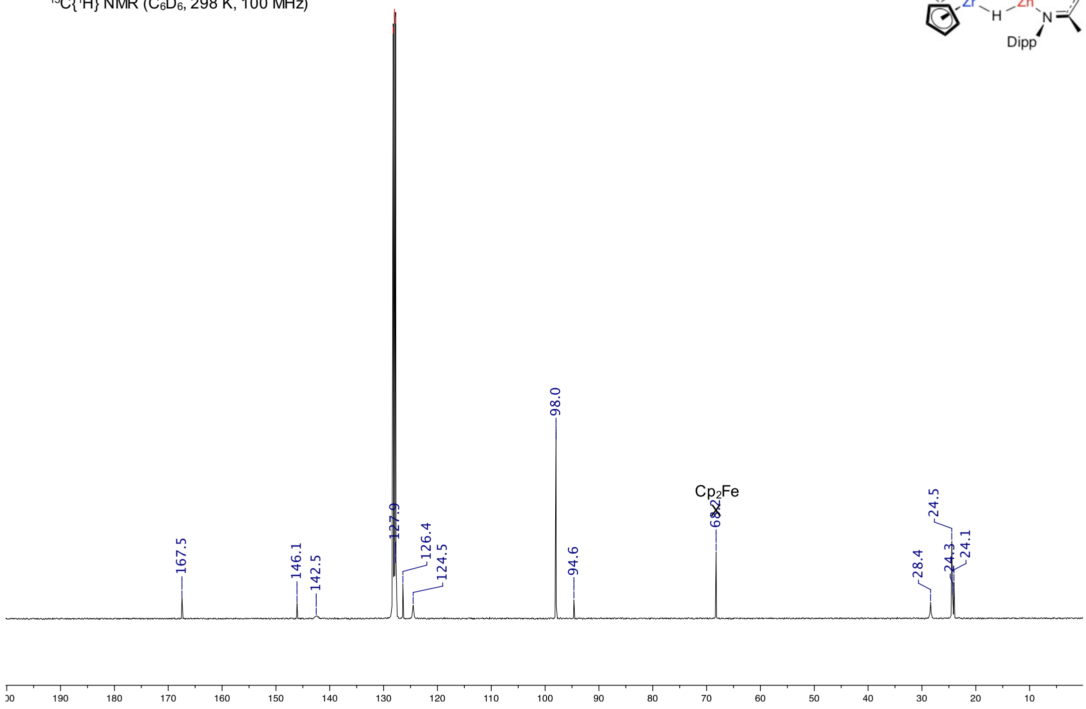

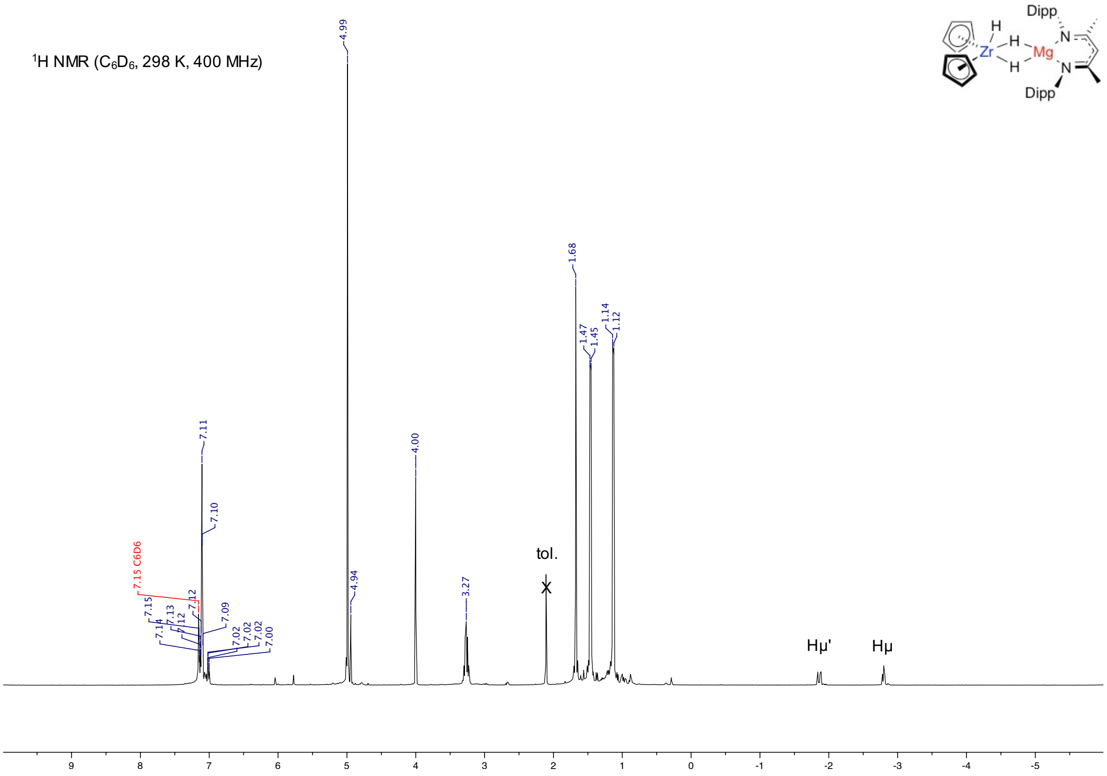
^{1}H NMR (C_6D_6, 298 K, 400 MHz)
Dipp
Zr
H
Mg
N
tol.
Hμ'
Hμ
7.15 C6D6
4.99
4.94
4.00
3.27
1.68
1.47
1.45
1.14
1.12
7.11
7.10

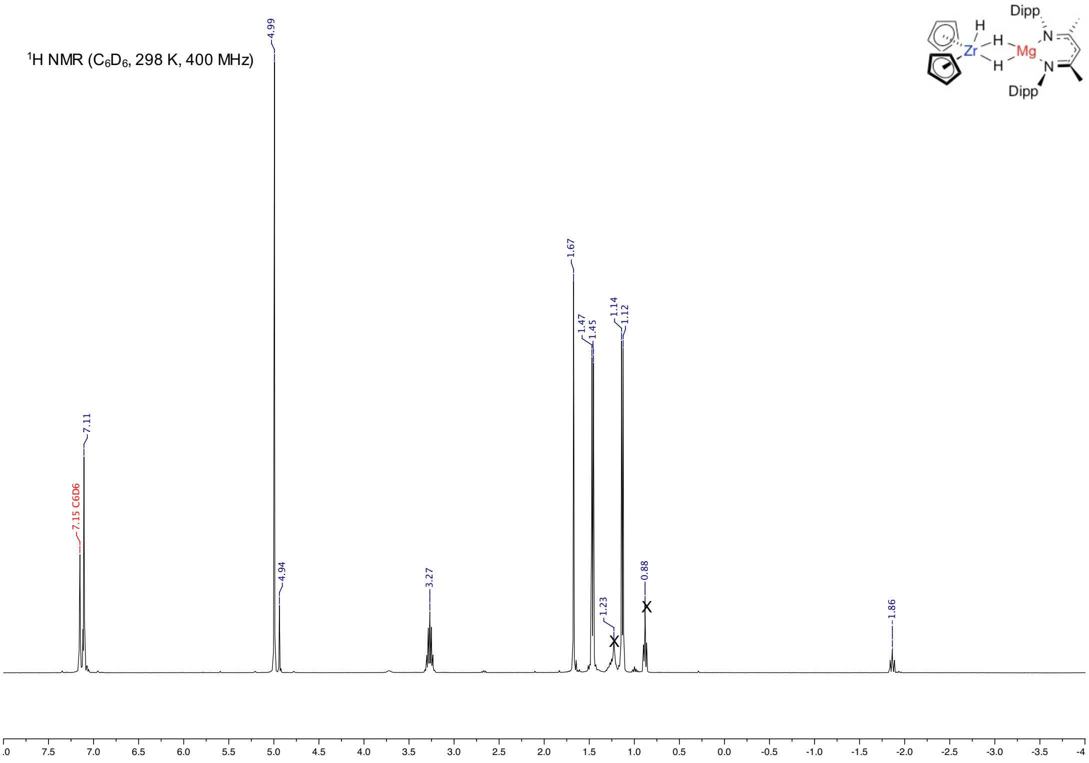
^{1}H NMR (C_6D_6, 298 K, 400 MHz)
Dipp
Zr
H
Mg
N
7.15 C6D6
7.11
4.99
4.94
3.27
1.67
1.47
1.45
1.14
1.12
1.23
0.88
-1.86

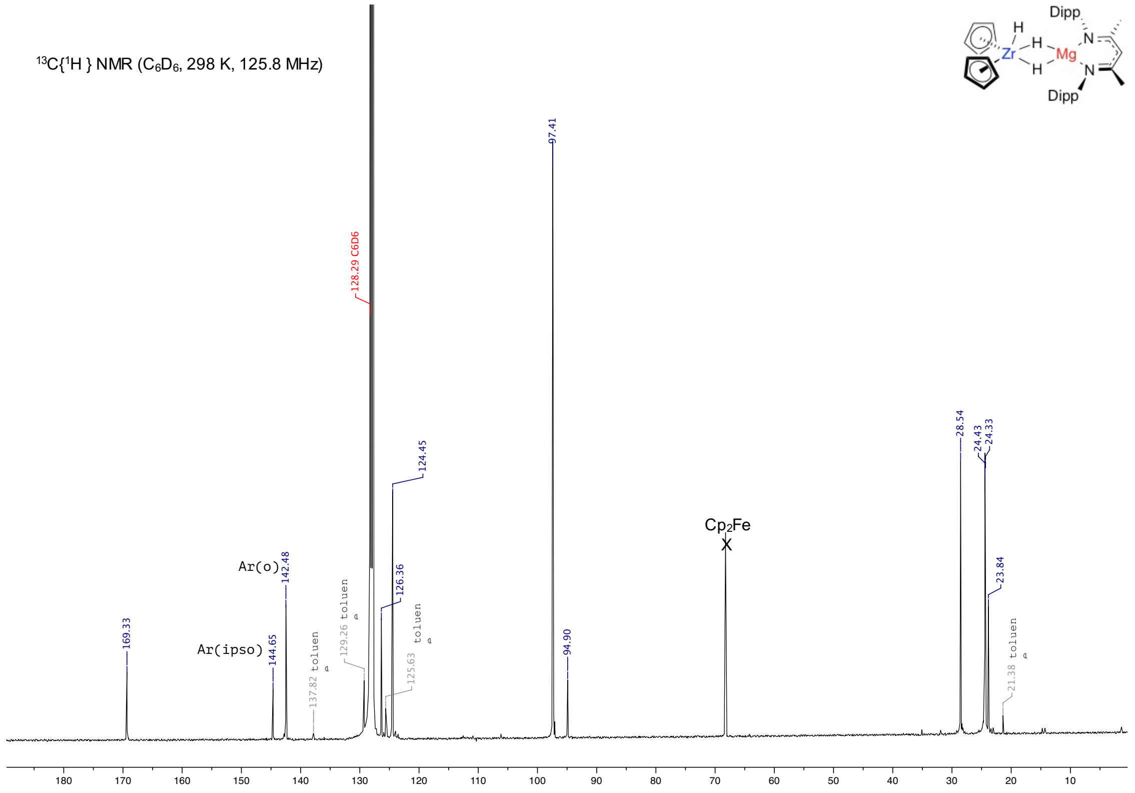

$^{13}C\{^1H\}$ NMR (C_6D_6, 298 K, 125.8 MHz)
Dipp
H
Zr
H
H
Mg
N
N
Dipp
169.33
Ar(ipso)
144.65
Ar(o)
142.48
137.82 toluene
129.26 toluene
128.29 C6D6
126.36
125.63 toluene
124.45
97.41
94.90
Cp2Fe
28.54
24.43
24.33
23.84
21.38 toluene
180 170 160 150 140 130 120 110 100 90 80 70 60 50 40 30 20 10

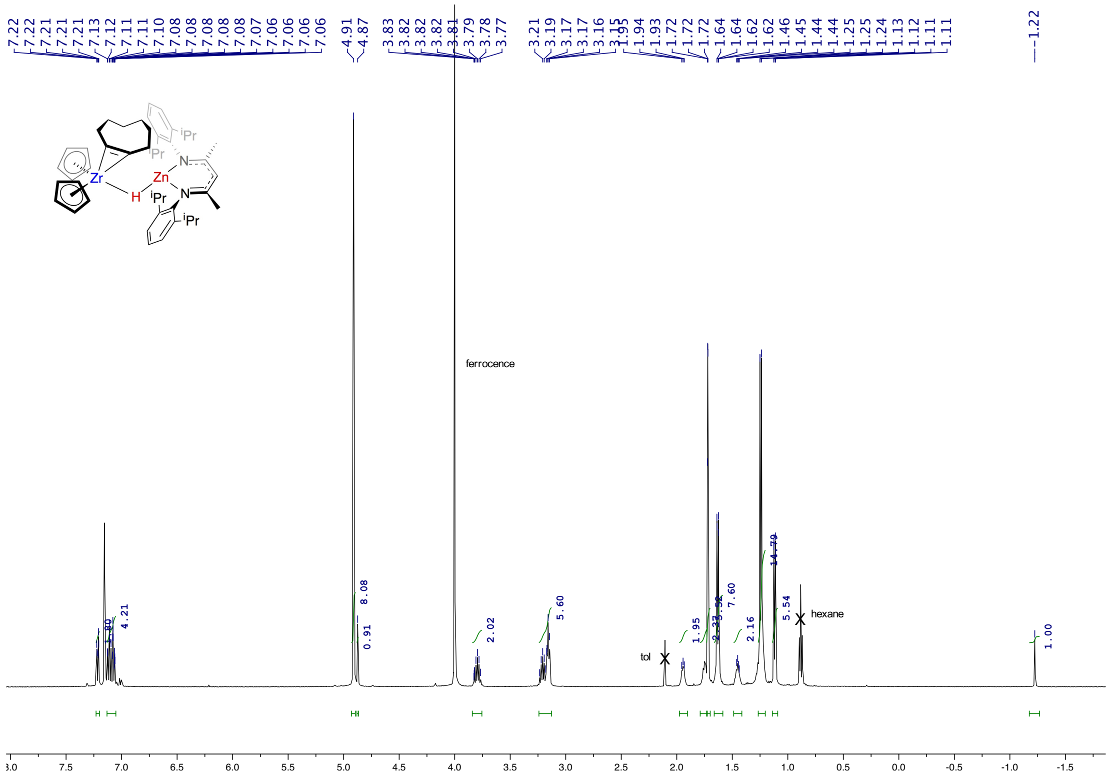
7.22 7.22 7.21 7.21 7.13 7.12 7.11 7.11 7.10 7.08 7.08 7.08 7.08 7.07 7.06 7.06 7.06 7.06
4.91 4.87
3.83 3.82 3.82 3.82 3.81 3.79 3.78 3.77
3.21 3.19 3.17 3.17 3.16 3.15
1.95 1.94 1.93 1.72 1.72 1.72 1.64 1.64 1.62 1.46 1.45 1.44 1.25 1.25 1.24 1.13 1.12 1.11 1.11
-1.22
Zr
Zn
H
N
iPr
ferrocence
tol
hexane

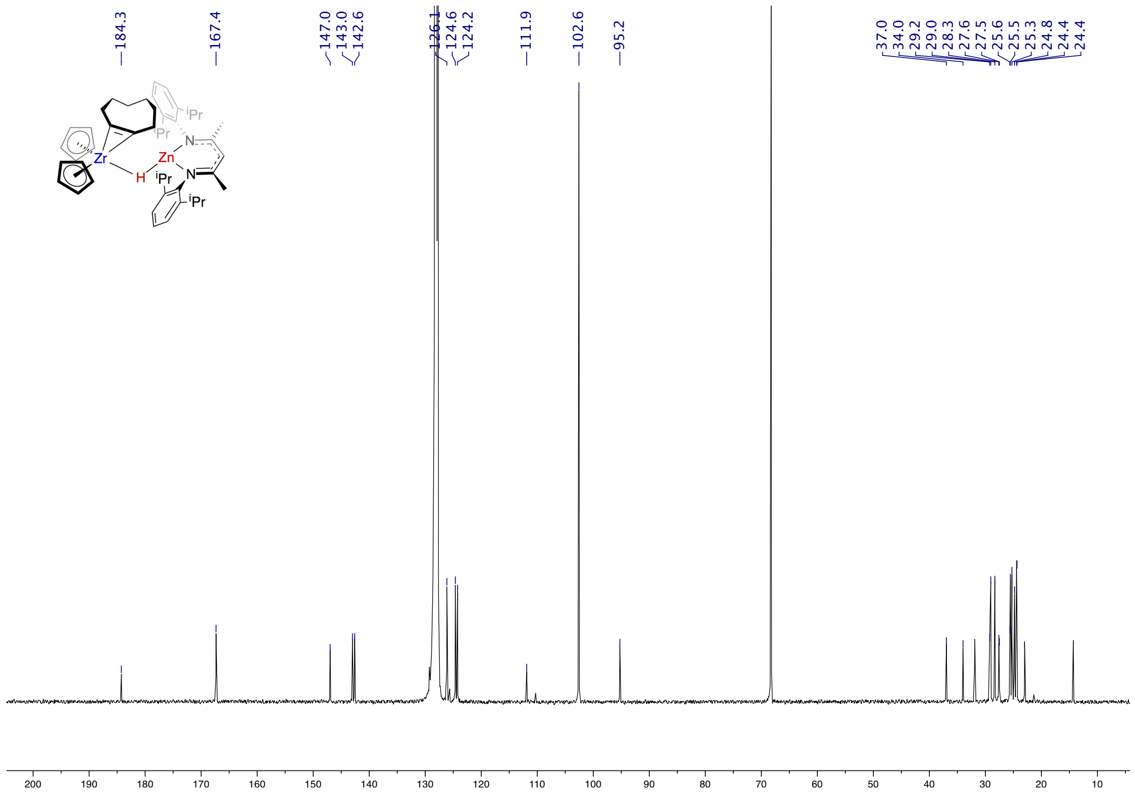
184.3
167.4
147.0 143.0 142.6
126.1 124.6 124.2
111.9
102.6
95.2
37.0 34.0 29.2 29.0 28.3 27.6 27.5 25.6 25.5 25.3 24.8 24.4 24.4
Zr
Zn
H
N
iPr

7 Z-Matrices

Zn•Zr

Sum of electronic and zero-point Energies= -1738.282729
Sum of electronic and thermal Energies= -1738.236378
Sum of electronic and thermal Enthalpies= -1738.235434
Sum of electronic and thermal Free Energies= -1738.362150
Lowest energy vibrations: 17.2, 34.8, 45.8 cm^{-1}

0 1

Zr	-0.11791600	2.58105200	0.11770600
N	-1.44536100	-1.85118400	0.01061500
C	-1.20693300	-3.16613000	-0.01768100
C	0.07578200	-3.73828000	-0.11804000
H	0.10131000	-4.81907800	-0.14438500
C	1.32831200	-3.10360000	-0.15215600
N	1.51343600	-1.77726900	-0.12759300
C	-2.37891000	-4.12565100	0.05536200
H	-2.95952300	-4.09726300	-0.87388400
H	-3.06244900	-3.84690400	0.86148800
H	-2.03441000	-5.14696600	0.21592800
C	2.55203200	-3.99389600	-0.20155700
H	3.17257200	-3.83847900	0.68812000
H	3.17297800	-3.74487200	-1.06804300
H	2.27205600	-5.04531300	-0.25572800
C	-2.78135500	-1.35173900	0.17260700
C	-3.64133400	-1.27018300	-0.94386200
C	-4.91140200	-0.71047700	-0.76926600
H	-5.58457600	-0.63560600	-1.61599900
C	-5.31948600	-0.23310700	0.47486800
H	-6.30479500	0.20323300	0.59210600
C	-4.45552200	-0.30765500	1.56408100
H	-4.77800600	0.06959600	2.52845000
C	-3.17641600	-0.86454300	1.43736000
C	-3.16260000	-1.68506700	-2.32832900
H	-2.37184000	-2.43280800	-2.20929900
C	-2.53836700	-0.47097400	-3.04091300
H	-1.70144200	-0.06316800	-2.46186400
H	-3.28811900	0.32054100	-3.15887700
H	-2.16802300	-0.75071200	-4.03296400
C	-4.26987700	-2.30739800	-3.18984100
H	-4.78029000	-3.11821500	-2.66035200
H	-3.84230600	-2.71115500	-4.11252700
H	-5.02023100	-1.56339400	-3.47647500
C	-2.26402800	-0.97321700	2.65218600
H	-1.23884300	-1.11031700	2.29051200
C	-2.62935500	-2.21255900	3.49026700
H	-2.52233200	-3.13335100	2.90875700
H	-3.66648200	-2.14507800	3.83769900
H	-1.97725400	-2.28433900	4.36684300
C	-2.27529200	0.29162800	3.52091200
H	-1.52263900	0.20126400	4.31096400
H	-3.24623000	0.44104800	4.00561700
H	-2.02927400	1.17482600	2.92479800
C	2.83862700	-1.22188700	-0.11863800
C	3.49545400	-1.01400900	1.11429400
C	4.72575300	-0.34779900	1.11135800
H	5.23622400	-0.16877500	2.05217700
C	5.29601400	0.10480200	-0.07674000
H	6.23954900	0.63779400	-0.05845100
C	4.65467300	-0.14068700	-1.28786800
H	5.11258400	0.19139300	-2.21487100
C	3.43108500	-0.81976800	-1.33410400
C	2.87725000	-1.46819300	2.43039000
H	2.06057900	-2.16100400	2.19870500
C	3.88921300	-2.21580500	3.31387200
H	3.38396800	-2.63671300	4.18859600
H	4.67252300	-1.54281300	3.67802700
H	4.37302200	-3.03154500	2.76732800
C	2.27344300	-0.27713900	3.19868600
H	1.48925900	0.22458400	2.62110800
H	3.05206900	0.46096300	3.42753100
H	1.83688700	-0.61587300	4.14448100
C	2.80210100	-1.15115300	-2.68179100
H	1.91108600	-1.76094600	-2.49482700
C	2.35655100	0.10838100	-3.44386200
H	1.94531000	-0.16633300	-4.42107100
H	3.20830500	0.77800200	-3.61439400
H	1.58835500	0.65464500	-2.88862800
C	3.76967300	-1.97669300	-3.54967800
H	4.12173800	-2.86827600	-3.02219500
H	4.64762600	-1.38371000	-3.82769100
H	3.27324000	-2.29373000	-4.47218700
C	2.38979600	2.41148100	0.60930800
H	2.81807400	1.50065300	0.99803200
C	2.30246900	2.76100800	-0.77116300
H	2.62603600	2.14413900	-1.59389000
C	1.73192000	4.05529600	-0.86122800
H	1.54325000	4.60405500	-1.77136800
C	1.47793800	4.51925200	0.46705500
H	1.06840500	5.48130700	0.73551400
C	1.90408700	3.51123200	1.37594600
H	1.87021000	3.56920200	2.45138100
C	-2.59156800	2.22439100	-0.46187300
H	-3.03352800	1.24059400	-0.40619800
C	-2.57526900	3.18848200	0.58400500
H	-2.97248900	3.05053000	1.57685500
C	-1.94526500	4.36433200	0.08769800
H	-1.78330500	5.27726500	0.64026200
C	-1.60611900	4.13834100	-1.28016200
H	-1.13695700	4.85067900	-1.94194200
C	-1.99875600	2.81937200	-1.61980500
H	-1.88328500	2.35116100	-2.58474700
H	-0.22252800	0.90375800	1.15706700
H	0.12472800	1.06332100	-1.15053700
H	-0.41458100	2.61310700	1.91778500
Zn	0.00660000	-0.42787300	-0.06374400

Mg•Zr

Sum of electronic and zero-point Energies= -1673.717161
Sum of electronic and thermal Energies= -1673.671272
Sum of electronic and thermal Enthalpies= -1673.670328
Sum of electronic and thermal Free Energies= -1673.795165

Lowest energy vibrations: 23.3, 38.4 53.2 cm^{-1}

0 1

Zr	-0.17461600	2.60893300	0.10706200
Mg	0.02297600	-0.47858400	-0.08632900
N	-1.38768600	-1.92738800	0.00626000
C	-1.14548300	-3.24242700	-0.02528900
C	0.14260600	-3.80207600	-0.12890400
H	0.18358900	-4.88246700	-0.15646200
C	1.38262200	-3.14393900	-0.16078300
N	1.53466900	-1.81166800	-0.13969800
C	-2.31065000	-4.20911700	0.04584200
H	-2.89418300	-4.17734300	-0.88169100
H	-2.99337700	-3.93848900	0.85566000
H	-1.96071000	-5.22981100	0.19869300
C	2.62550000	-4.00652600	-0.20230000
H	3.24382800	-3.82414900	0.68373200
H	3.23891300	-3.75312000	-1.07341400
H	2.37088900	-5.06507300	-0.24288200
C	-2.72842200	-1.43584100	0.17006600
C	-3.59139400	-1.35808200	-0.94495600
C	-4.86589200	-0.80935400	-0.76812600
H	-5.54036800	-0.73868700	-1.61434000
C	-5.27660800	-0.33695600	0.47690600
H	-6.26545900	0.09067500	0.59609100
C	-4.40908600	-0.40239500	1.56369300
H	-4.73225200	-0.02637800	2.52826500
C	-3.12520500	-0.94782000	1.43448500
C	-3.11514300	-1.76682700	-2.33212900
H	-2.29793600	-2.48621600	-2.21702900
C	-2.54524100	-0.53745900	-3.06436500
H	-1.71482000	-0.09008200	-2.50483900
H	-3.32348900	0.22672300	-3.17716400
H	-2.18065000	-0.81246300	-4.05976800
C	-4.21167100	-2.43419800	-3.17398700
H	-4.68469000	-3.25948300	-2.63230100
H	-3.78373300	-2.82799800	-4.10080800
H	-4.99279200	-1.71966400	-3.45289900
C	-2.21111500	-1.04350700	2.64934300
H	-1.18424500	-1.17777800	2.28976000
C	-2.56731500	-2.28200000	3.49258600
H	-2.45587900	-3.20383900	2.91351700
H	-3.60431300	-2.21904100	3.84108000
H	-1.91363700	-2.34692200	4.36847800
C	-2.23083600	0.22620400	3.51101900
H	-1.47733500	0.14571700	4.30134400
H	-3.20249100	0.37016100	3.99583000
H	-1.99246000	1.10812900	2.90971100
C	2.84969600	-1.22889800	-0.11071200
C	3.47118800	-0.98142600	1.13352600
C	4.68421900	-0.28396600	1.14778600
H	5.16726600	-0.07604500	2.09729100
C	5.27234500	0.16104500	-0.03426400
H	6.20116900	0.71866400	-0.00325000
C	4.66825700	-0.12385900	-1.25588100
H	5.14015400	0.20290300	-2.17759300
C	3.46379900	-0.83485600	-1.31840700
C	2.83866700	-1.43523400	2.44307800
H	2.02872300	-2.13299000	2.20262600
C	3.84576700	-2.17706900	3.33750000
H	3.33542300	-2.59483200	4.21079600
H	4.62527800	-1.50031700	3.70297400
H	4.33497600	-2.99440500	2.79837200
C	2.22239400	-0.24680700	3.20551300
H	1.44264600	0.25588700	2.62210600
H	2.99583200	0.49367600	3.44366300
H	1.77722300	-0.58795900	4.14638000
C	2.86973000	-1.19958400	-2.67282100
H	2.01775100	-1.86549700	-2.49505200
C	2.35021700	0.03681900	-3.42603400
H	1.94602100	-0.25419800	-4.40141100
H	3.16394100	0.75172600	-3.59846500
H	1.56025000	0.54298400	-2.86223800
C	3.88996000	-1.95842300	-3.53987700
H	4.28931300	-2.83264200	-3.01668900
H	4.73401400	-1.31408800	-3.80761700
H	3.41934600	-2.29585600	-4.46863300
C	2.33937600	2.48122900	0.61418400
H	2.77700600	1.59018800	1.03748100
C	2.25502600	2.78037900	-0.77814000
H	2.58760600	2.13690500	-1.57695400
C	1.66812800	4.06312900	-0.91707900
H	1.47795800	4.57729600	-1.84692700
C	1.40485500	4.57211800	0.39336100
H	0.98405200	5.53907900	0.62451400
C	1.83757800	3.60222000	1.33919100
H	1.79857000	3.69794000	2.41165400
C	-2.64396000	2.19520700	-0.46519600
H	-3.06135100	1.20083400	-0.40258200
C	-2.64557400	3.16490600	0.57563200
H	-3.03365100	3.02281600	1.57148900
C	-2.04363600	4.35194900	0.07094500
H	-1.90007700	5.27099800	0.61846100
C	-1.70392200	4.12639000	-1.29691100
H	-1.25225100	4.84525200	-1.96390800
C	-2.06891900	2.79754000	-1.62827200
H	-1.94541400	2.32627600	-2.59075900
H	-0.25265600	0.93371700	1.11650500
H	0.08785300	1.10228600	-1.14572900
H	-0.46050700	2.65315600	1.91114900

Al•Zr

Sum of electronic and zero-point Energies= -
1361.400027
Sum of electronic and thermal Energies= -
1361.364445
Sum of electronic and thermal Enthalpies= -
1361.363501
Sum of electronic and thermal Free Energies= -
1361.466906

Lowest Energy Vibrations: 16.9, 38.2, 54.1 cm^{-1}

0 1

Al	0.30968900	0.72260500	-0.80935900
Zr	-0.33766000	-2.13430800	0.06165300
H	0.75351800	0.56069800	-2.36235800
H	-0.99701100	-0.64020700	-0.99331600
H	0.65870500	-0.49299100	0.42896100
H	0.93923600	-2.15805700	1.37816400
N	1.80780100	1.76835900	-0.05791000
C	1.81332600	3.09100500	0.05942300
C	0.67318400	3.88306500	-0.20213900
H	0.81136100	4.95450800	-0.16798600
C	-0.63295900	3.42465300	-0.36695200
N	-0.98067200	2.11717400	-0.43706100
C	3.06498400	3.78583800	0.54043100
H	3.27857900	3.49285600	1.57455700
H	2.94930500	4.86860700	0.50070500
H	3.93363900	3.49402800	-0.05563500
C	-1.73135200	4.46243600	-0.44452300
H	-2.38037200	4.26394100	-1.30286700
H	-1.30550800	5.46094500	-0.54125800
H	-2.36752400	4.43953500	0.44561700
C	2.97721200	0.99170300	0.27147500
C	3.09815700	0.44588800	1.56065600
C	4.18991600	-0.38681500	1.82908800
H	4.28778900	-0.82138600	2.81832700
C	5.13657800	-0.66532300	0.84621600
H	5.97339400	-1.31773500	1.06818600
C	5.01150100	-0.09799300	-0.42059100
H	5.75315400	-0.30283900	-1.18567300
C	3.93413400	0.73971800	-0.72922800
C	2.06788500	0.72924500	2.62230200
H	1.16752600	0.13048500	2.43772100
H	1.77125300	1.78310700	2.63326100
H	2.45300000	0.46720200	3.61066300
C	3.80890400	1.36389200	-2.09745600
H	3.91546500	2.45459800	-2.05484000
H	2.83018800	1.15643300	-2.53933700
H	4.58365100	0.97941400	-2.76469200
C	-2.36866500	1.77759000	-0.26551900
C	-3.15527000	1.43874400	-1.37856500
C	-4.50291700	1.12411800	-1.16965000
H	-5.12107700	0.86751400	-2.02370700
C	-5.05299600	1.14050600	0.11183100
H	-6.09807200	0.89277500	0.25753000
C	-4.25378800	1.46369800	1.20768200
H	-4.67323600	1.45913600	2.20844800
C	-2.90353600	1.78622900	1.03552500
C	-2.55435800	1.41661000	-2.75931000
H	-2.00004100	2.33663500	-2.97180300
H	-3.33103300	1.29552500	-3.51776200
H	-1.84277300	0.58870800	-2.86187200
C	-2.02580200	2.13846100	2.21148700
H	-1.81495600	3.21401600	2.25739600
H	-1.05530800	1.63527700	2.13689300
H	-2.50457800	1.85206500	3.15123600
C	1.60236100	-2.35164900	-1.58507100
H	2.32140500	-1.55126400	-1.67802800
C	0.41720000	-2.51108600	-2.36348800
H	0.07196000	-1.82783000	-3.12363800
C	-0.22762600	-3.70208300	-1.93898000
H	-1.14494600	-4.10522000	-2.34034900
C	0.56977500	-4.29265300	-0.90831500
H	0.36167500	-5.22230400	-0.40094800
C	1.70713200	-3.46529600	-0.70021600
H	2.50884300	-3.64169600	-0.00259700
C	-1.97993300	-1.44608500	1.89447300
H	-1.86640000	-0.49647500	2.39275500
C	-1.41628500	-2.68456000	2.32337500
H	-0.78820900	-2.83147000	3.18650300
C	-1.83658600	-3.68951300	1.41181900
H	-1.59094400	-4.73901300	1.46932500
C	-2.68306200	-3.07701300	0.43426300
H	-3.18163700	-3.58323100	-0.37837800
C	-2.78338500	-1.69625300	0.74240200
H	-3.35183100	-0.95904700	0.19647300

Compound 1 Molecule A
wB97x / 6311G+(d,p) / SDD

ZPE = -2211.744639
E= -2211.691672
H= -2211.690728
G= -2211.827310

0 1

Zr	0.24892300	-1.63491000	-1.65242100
Zn	-0.11917900	0.38212200	0.26994000
H	-0.07285600	0.30524700	-1.46252400
N	-1.79597900	1.15181500	1.07320100
C	-1.72087600	1.99454400	2.11224600
C	-0.52690700	2.54721500	2.60445900
H	-0.64260100	3.20625900	3.45312600
C	0.75773800	2.54950000	2.02736400
N	1.14925100	1.74262200	1.03637300
C	-2.99427200	2.45203700	2.79323300
H	-3.72685700	2.80286300	2.06562100
H	-2.78874800	3.25497800	3.49684100
H	-3.45781100	1.62949700	3.33953700
C	1.69153000	3.62274800	2.55112600
H	2.74041000	3.37307800	2.41771800
H	1.50121400	3.80259900	3.60846000
H	1.50457900	4.55887800	2.01948600
C	-3.08984600	0.83621000	0.51153300
C	-3.49317100	1.50601800	-0.66951400
C	-4.73007600	1.18075200	-1.23076400
H	-5.05574400	1.67729300	-2.13395400
C	-5.55541400	0.22008100	-0.65784200
H	-6.50730300	-0.01976700	-1.11387100
C	-5.14730700	-0.43244100	0.49433800
H	-5.78691900	-1.18836900	0.93319200
C	-3.91986100	-0.14040700	1.09881300
C	-2.60840100	2.56463400	-1.32639500
H	-1.57920800	2.19858100	-1.27736800
C	-2.94180800	2.79954100	-2.80554200
H	-3.90221700	3.30738400	-2.92534300
H	-2.97985600	1.86768300	-3.37229400
H	-2.18074500	3.43653200	-3.25876100
C	-2.66201200	3.91022200	-0.58170300
H	-3.68978700	4.28120200	-0.53672700
H	-2.05872600	4.65456200	-1.10971700
H	-2.27585100	3.83659800	0.43455800
C	-3.52204900	-0.92954800	2.34010400
H	-2.63730200	-0.45850600	2.77143700
C	-4.62112800	-0.92897000	3.41454000
H	-4.95990800	0.08172100	3.65079100
H	-4.24798800	-1.38852800	4.33253600
H	-5.49301500	-1.50254400	3.09204100
C	-3.14892700	-2.37464100	1.96948200
H	-4.00491600	-2.88795300	1.52250400
H	-2.84853200	-2.93148600	2.86100900
H	-2.32324800	-2.40260800	1.25610900
C	2.40369900	2.01612700	0.37148200
C	3.57653700	1.30845000	0.72640600
C	4.76164100	1.61722600	0.05380200
H	5.67189900	1.09202300	0.30540800
C	4.80036400	2.58027600	-0.94874400
H	5.73177600	2.79659600	-1.45603400
C	3.63947500	3.25021500	-1.30005800
H	3.66737600	3.99202900	-2.08927500
C	2.42910000	2.98537300	-0.65115100
C	3.56123600	0.21350700	1.79365600
H	2.68369700	-0.40448300	1.58376100
C	3.41565900	0.75462200	3.22692900
H	4.20746500	1.47571900	3.44790700
H	3.50312300	-0.06752800	3.94200700
H	2.45343900	1.23341100	3.39642300
C	4.79455700	-0.69963600	1.74917100
H	5.69241900	-0.16988300	2.07789200
H	4.98566200	-1.10102900	0.75239300
H	4.64737400	-1.54192600	2.42770400
C	1.18358400	3.74334400	-1.09762700
H	0.35179600	3.44615900	-0.45572200
C	0.80964800	3.37777200	-2.54268900
H	1.61640700	3.63602500	-3.23361400
H	-0.08241800	3.92712700	-2.85336900
H	0.60510100	2.30936500	-2.64004400
C	1.35730400	5.26433700	-0.96289100
H	2.11924400	5.63794200	-1.65153700
H	1.65544700	5.54783800	0.04800600
H	0.41945700	5.77419600	-1.19686600
C	0.24591800	-1.73964700	0.79253700
C	0.38687000	-2.87722000	0.12724300
C	0.56081100	-4.31694800	0.52722000
H	0.42799400	-4.96058100	-0.34872400
H	-0.23072500	-4.60713000	1.22546500
C	1.93886500	-4.64638000	1.14905000
H	1.86986800	-5.61410500	1.65901800
H	2.66507600	-4.78062300	0.34040300
C	2.50438300	-3.59709000	2.10918900
H	3.50927100	-3.91827200	2.40582600
H	2.63495100	-2.65904700	1.56351100
C	1.69812400	-3.33163400	3.38635000
H	2.28100300	-2.63991500	4.00804200
H	1.62025600	-4.26237800	3.96125500
C	0.29047300	-2.73826000	3.21260600
H	-0.40902300	-3.49605300	2.85864000
H	-0.06734500	-2.44784800	4.20713800
C	0.20791200	-1.49867300	2.30606000
H	1.00633100	-0.80373500	2.59374100
H	-0.71552000	-0.96554300	2.55084100
C	-2.31462400	-1.81455400	-1.74045300
H	-2.91491100	-1.40364100	-0.94714000
C	-1.83382900	-3.14269900	-1.82505800
H	-1.97463100	-3.91672900	-1.08920300
C	-1.12583400	-3.27523000	-3.04706600
H	-0.66136900	-4.17642100	-3.41381200
C	-1.21272600	-2.03406700	-3.74202300
H	-0.79780500	-1.82373200	-4.71418800
C	-1.93900100	-1.13320900	-2.93463200
H	-2.18696900	-0.11414800	-3.17873100
C	2.47717200	-0.44353500	-2.30765300
H	2.62249200	0.60490500	-2.10303600
C	1.92795200	-0.99809000	-3.49582900
H	1.58217600	-0.44088000	-4.35140500
C	1.96667500	-2.41104700	-3.37595400
H	1.65971700	-3.11867400	-4.12834100
C	2.51681300	-2.73252800	-2.10602100
H	2.70249500	-3.72361700	-1.72663400
C	2.83685200	-1.51201300	-1.45518100
H	3.26997200	-1.41661700	-0.47541000

Compound 1 Molecule B
wB97x 6311G+(d,p) / SDD

ZPE= -2211.744294
E= -2211.690880
H= -2211.689936
G= -2211.828099

0 1

Zr	0.10981000	1.64432100	-1.64318500
Zn	-0.03253100	-0.41920700	0.27733100
H	0.06950800	-0.32123200	-1.45224600
N	-1.64997700	-1.31693400	1.09621500
C	-1.50265300	-2.14939000	2.13633000
C	-0.26864600	-2.61494300	2.62374200
H	-0.33317200	-3.26527400	3.48348400
C	1.00866800	-2.51944500	2.04454600
N	1.31335300	-1.71685200	1.01959700
C	-2.73136500	-2.70148800	2.82900900
H	-3.25224300	-1.91557000	3.37636700
H	-2.45810000	-3.48342700	3.53313200
H	-3.44020600	-3.11144600	2.10890200
C	2.07025700	-3.43755900	2.61176300
H	1.64470700	-4.11243200	3.35010200
H	2.86722200	-2.86519000	3.08803600
H	2.53524700	-4.03164100	1.82382000
C	-2.97768700	-1.10362700	0.56116300
C	-3.36096300	-1.81712100	-0.60175600
C	-4.63814700	-1.60293200	-1.12431100
H	-4.94956100	-2.13351900	-2.01289300
C	-5.52358800	-0.70990300	-0.53211300
H	-6.50656200	-0.55663100	-0.95847200
C	-5.13469900	-0.01245900	0.59954100
H	-5.82128600	0.69178100	1.05332600
C	-3.86799900	-0.19335200	1.16606000
C	-2.41135700	-2.80145600	-1.28435600
H	-1.42405600	-2.33043600	-1.30592500
C	-2.80070400	-3.11674900	-2.73536500
H	-3.71058300	-3.72060500	-2.78299700
H	-2.00529900	-3.69308700	-3.21103100
H	-2.96390300	-2.21585800	-3.32927200
C	-2.28451500	-4.12650100	-0.51178400
H	-3.26779000	-4.58762800	-0.38377600
H	-1.83189300	-3.99511500	0.46998400
H	-1.65665100	-4.82569000	-1.07104200
C	-3.50673600	0.64230900	2.38859700
H	-2.55137400	0.28422500	2.77659600
C	-4.55143500	0.52026200	3.51060700
H	-5.49155600	0.99980300	3.22857600
H	-4.18976900	1.01449100	4.41506300
H	-4.77358000	-0.52001600	3.75499400
C	-3.33225400	2.12004700	2.00150400
H	-4.25311300	2.51338800	1.56206900
H	-2.52512100	2.24793700	1.27831100
H	-3.09993900	2.71936700	2.88515200
C	2.58133000	-1.87100200	0.34453900
C	3.74949000	-1.25648500	0.83843500
C	4.93354000	-1.37449800	0.10462000
H	5.83327500	-0.89435300	0.46974200
C	4.97692800	-2.08813700	-1.08530400
H	5.90176400	-2.16318100	-1.6426430
C	3.82611300	-2.71188900	-1.54980200
H	3.86545900	-3.28482700	-2.46846000
C	2.62061600	-2.62324900	-0.84957400
C	3.76149000	-0.43184500	2.11766700
H	2.79548200	-0.56001000	2.61127700
C	4.85993700	-0.87580500	3.09532100
H	4.80928900	-1.94571100	3.30648000
H	4.76427200	-0.33591200	4.04007800
H	5.85439700	-0.66550000	2.69522000
C	3.91448900	1.05731800	1.78315500
H	4.87450000	1.25069400	1.29690000
H	3.86184300	1.66888100	2.68766900
H	3.12263000	1.37729800	1.10542500
C	1.40776900	-3.38845300	-1.36423700
H	0.54741500	-3.09357800	-0.76167700
C	1.08718000	-3.06247000	-2.82849500
H	0.88437800	-1.99736200	-2.95350500
H	0.20322000	-3.61698100	-3.15279100
H	1.90927600	-3.33949100	-3.49309300
C	1.60400600	-4.90195900	-1.17558600
H	2.46231300	-5.25913600	-1.75080100
H	0.71965000	-5.44766500	-1.51400700
H	1.77415800	-5.15315300	-0.12658500
C	0.10891800	1.74654300	0.79578200
C	0.00664600	2.89072000	0.13321000
C	-0.11543800	4.33741000	0.53027300
H	-0.92952400	4.45199300	1.25365500
H	-0.41299800	4.93131900	-0.34016500
C	1.17116800	4.96692600	1.10597000
H	1.84510900	5.20450600	0.27505000
H	0.91281600	5.92455200	1.57228900
C	1.94322000	4.09742900	2.09974000
H	2.28555500	3.20110000	1.58232100
H	2.84753700	4.64110200	2.39527600
C	1.20617600	3.67729300	3.37728800
H	1.93750200	3.18263100	4.02840900
H	0.87993400	4.57389300	3.91825500
C	0.00176700	2.73231000	3.21786300
H	-0.87457500	3.28215500	2.87756300
H	-0.25390500	2.36120600	4.21703100
C	0.22197900	1.51032300	2.30551700
H	1.20867000	1.08054900	2.52207000
H	-0.49001200	0.73434500	2.60832900
C	-2.46745800	1.54662800	-1.71338600
H	-3.02004400	1.18103200	-0.86550200
C	-2.11252300	2.89549600	-1.96389800
H	-2.33311000	3.73731600	-1.32907600
C	-1.42811000	2.94300700	-3.20574100
H	-1.05175300	3.83099600	-3.68778900
C	-1.39218300	1.62183300	-3.73868200
H	-0.96711800	1.32908500	-4.68446500
C	-2.03210800	0.76435200	-2.81760100
H	-2.18010200	-0.29537700	-2.93313400
C	2.64635800	1.12619700	-1.81544000
H	3.08335400	0.34611400	-1.21389300
C	2.10050700	0.95455100	-3.12418200
H	2.05118700	0.02357200	-3.66554800
C	1.69713500	2.22249700	-3.59522000
H	1.26854400	2.43482700	-4.56036600
C	1.95925300	3.17889800	-2.57374000
H	1.78257500	4.24099500	-2.63608100
C	2.58209400	2.50015900	-1.49611000
H	2.91723300	2.95702200	-0.580

Cyclooctyne
B3LYP / 6311G+(d,p)

Sum of electronic and zero-point Energies= -311.907271
Sum of electronic and thermal Energies= -311.899441
Sum of electronic and thermal Enthalpies= -311.898497
Sum of electronic and thermal Free Energies= -311.939077

Lowest Energy vibrations = 152.0, 214.1. 353.0 cm^{-1}

C	-0.60088700	-1.48204800	-0.03781000
C	0.60164800	-1.48238800	0.03780300
C	-0.69811700	1.38152700	-0.35236400
C	1.94460800	-0.92237100	0.13362300
C	0.69755100	1.38181600	0.35243100
C	1.85217800	0.57020100	-0.28906300
H	2.31141200	-0.99899500	1.16402100
H	-1.03690900	2.42191200	-0.38603200
H	0.57966600	1.07897500	1.39940200
H	-0.58029600	1.07845000	-1.39927000
H	2.66974000	-1.44710500	-0.49639500
H	1.03594200	2.42232900	0.38589900
H	2.80568800	1.04881600	-0.03893800
H	1.75853700	0.61746600	-1.37939200
C	-1.94420800	-0.92301300	-0.13359300
H	-2.31108800	-0.99970000	-1.16394600
H	-2.66876300	-1.44819200	0.49666300
C	-1.85251300	0.56974900	0.28903600
H	-1.75947300	0.61720000	1.37939300
H	-2.80602100	1.04800300	0.03821700

1,2-Cyclooctadiene
B3LYP / 6311G+(d,p)

Sum of electronic and zero-point Energies= -311.910979
Sum of electronic and thermal Energies= -311.903332
Sum of electronic and thermal Enthalpies= -311.902388
Sum of electronic and thermal Free Energies= -311.942439

Lowest Energy vibrations = 207.8, 267.4. 299.2 cm^{-1}

C	-0.32730700	-1.54464900	0.18972100
C	0.95724900	-1.46098700	0.41177000
H	1.42110600	-1.85544400	1.31232200
C	-1.52987200	-1.18977600	-0.17884000
H	-2.16278400	-1.84844600	-0.76799900
C	-2.00416600	0.23407500	0.09632300
H	-2.94338800	0.39673000	-0.44144000
H	-2.23386100	0.37153700	1.15918000
C	1.74992000	-0.55843400	-0.51597700
H	2.80283600	-0.85323600	-0.56692100
H	1.34301600	-0.65546400	-1.52524600
C	-0.97958200	1.30946000	-0.35149400
H	-1.49883800	2.27374100	-0.33029100
H	-0.72158400	1.13266900	-1.40174700
C	1.65864300	0.92929200	-0.06508800
H	2.41451300	1.11004800	0.70840000
H	1.94978300	1.55389200	-0.91812800
C	0.31215200	1.43429900	0.50768600
H	0.13969900	0.95843100	1.47722400
H	0.46728200	2.49586700	0.73004100

1,3-Cyclooctadiene

B3LYP / 6311G+(d,p)

Sum of electronic and zero-point Energies= -311.945741
Sum of electronic and thermal Energies= -311.938371
Sum of electronic and thermal Enthalpies= -311.937427
Sum of electronic and thermal Free Energies= -311.976916

Lowest Energy vibrations = 227.5 248.2. 357.8 cm^{-1}

C	1.70098700	-0.76222900	-0.07402900
C	1.70052300	0.76317900	0.07401000
C	-1.74288200	-0.64143100	-0.35450100
C	0.48225900	1.49208200	-0.54513000
C	-1.74328800	0.64044600	0.35447000
C	-0.75756200	1.54949800	0.31142100
H	0.77906600	2.52150100	-0.76880400
H	0.24384000	1.01757300	-1.50335600
H	-2.63461800	0.87588400	0.93378200
H	-0.87913900	2.44969400	0.91038300
C	0.48320200	-1.49189100	0.54520000
C	-0.75655900	-1.54981800	-0.31136700
H	-2.63393500	-0.87755200	-0.93394700
H	-0.87737900	-2.45005500	-0.91037100
H	1.78005800	1.02875700	1.13493000
H	2.60764000	1.14628300	-0.40593500
H	2.60841400	-1.14487200	0.40570800
H	1.78052500	-1.02755800	-1.13500900
H	0.24471200	-1.01755000	1.50349500
H	0.78073500	-2.52113300	0.76868200

1,4-Cyclooctadiene
B3LYP / 6311G+(d,p)

Sum of electronic and zero-point Energies= -311.938067
Sum of electronic and thermal Energies= -311.930453
Sum of electronic and thermal Enthalpies= -311.929509
Sum of electronic and thermal Free Energies= -311.971413

Lowest Energy vibrations = 14.1, 336.8, 360.7 cm^{-1}

C	1.91446100	-0.00110900	0.00222400
C	1.18473700	1.25519900	0.43253300
C	-1.04272800	-1.02596300	0.77904600
C	-0.02236800	1.71374800	0.08938200
C	-1.89720100	-0.00006900	0.00206000
C	-1.04499500	1.02551400	-0.77799100
H	2.59031800	-0.28654800	0.82165100
H	-2.55005900	0.52023800	0.71185500
H	2.59962400	0.28208100	-0.81013200
H	1.78110200	1.89075400	1.08479900
H	-1.70613800	-1.77345100	1.22435300
H	-0.32356600	2.66748700	0.51824400
H	-2.55230900	-0.52020900	-0.70580500
H	-1.71006900	1.77286500	-1.22137500
H	-0.55071500	0.51466300	-1.60747800
C	1.18396900	-1.25449500	-0.43539100
H	1.77885900	-1.88715700	-1.09172000
C	-0.02327900	-1.71300600	-0.09282200
H	-0.32714300	-2.66407700	-0.52554100
H	-0.54548300	-0.51556200	1.60689700

1,5-Cyclooctadiene

B3LYP / 6311G+(d,p)

Sum of electronic and zero-point Energies= -311.937397
Sum of electronic and thermal Energies= -311.929943
Sum of electronic and thermal Enthalpies= -311.928999
Sum of electronic and thermal Free Energies= -311.968904

Lowest Energy vibrations = 121.1, 323.5, 336.1 cm^{-1}

C	0.00813200	-1.70426100	-0.21645700
C	1.22516700	-1.22668000	-0.49330900
H	-0.26306100	-2.63820000	-0.70479500
H	1.83463400	-1.82824700	-1.16608400
C	-1.07155700	-1.11535300	0.66417800
H	-1.75144800	-1.92243300	0.95479500
H	-0.65915200	-0.73101400	1.59724100
C	1.91970100	0.02697400	-0.02624900
H	2.41468100	0.47333300	-0.89844400
H	2.73861000	-0.26045800	0.64864600
C	1.07156800	1.11537600	0.66411800
H	0.65918500	0.73113900	1.59721900
H	1.75155200	1.92241000	0.95463900
C	-1.91967800	-0.02704900	-0.02628600
H	-2.73877900	0.26036700	0.64839200
H	-2.41445000	-0.47341500	-0.89859700
C	-0.00815300	1.70429800	-0.21650200
C	-1.22518100	1.22670600	-0.49318300
H	-1.83463300	1.82814000	-1.16610000
H	0.26285900	2.63831500	-0.7047770
